# Synthesis, Characterization and Antimicrobial Activity of Multiple Morphologies of Gold/Platinum Doped Bismuth Oxide Nanostructures

**DOI:** 10.3390/ijms241713173

**Published:** 2023-08-24

**Authors:** Cătălin Ianăși, Nicoleta Sorina Nemeş, Bogdan Pascu, Radu Lazău, Adina Negrea, Petru Negrea, Narcis Duteanu, Mihaela Ciopec, Jiri Plocek, Popa Alexandru, Bianca Bădescu, Daniel Marius Duda-Seiman, Delia Muntean

**Affiliations:** 1 Faculty of Industrial Chemistry and Environmental Engineering, Politehnica University Timişoara, 2 Victoriei Square, 300006 Timisoara, Romania; cianasic@yahoo.com (C.I.); adina.negrea@upt.ro (A.N.);; 2 Research Institute for Renewable Energies-ICER, Politehnica University Timisoara, 138 Gavril Musicescu Street, 300501 Timisoara, Romania; 3 Institute of Inorganic Chemistry, Academy of Sciences of the Czech Republic, v.v.i, Husinec-Řež 1001, 25068 Řež, Czech Republic; 4 “Coriolan Dragulescu” Institute of Chemistry, Romanian Academy, 24 Mihai Viteazu Bvd., 300223 Timisoara, Romania; 5 Doctoral School, “Victor Babeș” University of Medicine and Pharmacy, 2 Eftimie Murgu Street, 300041 Timisoara, Romania; 6 Department of Cardiology, “Victor Babeș” University of Medicine and Pharmacy, 2 Eftimie Murgu Street, 300041 Timisoara, Romania; 7 Multidisciplinary Research Centre on Antimicrobial Resistance, Department of Microbiology, “Victor Babeş” University of Medicine and Pharmacy, 2 Eftimie Murgu Street, 300041 Timișoara, Romania

**Keywords:** bismuth oxide, sol-gel, gold, platinum, antimicrobial activity, structural morphology

## Abstract

Bismuth oxides were synthesized from bismuth carbonate using the sol-gel method. Studies have described the formation of Bi_2_O_3_, as a precursor of HNO_3_ dissolution, and intermediate oxides, such as Bi_x_O_y_ when using H_2_SO_4_ and H_3_PO_4_. The average size of the crystallite calculated from Scherrer’s formula ranged from 9 to 19 nm, according to X-ray diffraction. The FTIR analysis showed the presence of specific Bi_2_O_3_ bands when using HNO_3_ and of crystalline phases of “bismuth oxide sulphate” when using H_2_SO_4_ and “bismuth phosphate” when using H_3_PO_4_. The TG curves showed major mass losses and specific thermal effects, delimited in four temperature zones for materials synthesized with HNO_3_ (with loss of mass between 24% and 50%) and H_2_SO_4_ (with loss of mass between 45% and 76%), and in three temperature zones for materials synthesized with H_3_PO_4_ (with loss of mass between 13% and 43%). Further, the thermal stability indicates that materials have been improved by the addition of a polymer or polymer and carbon. Confocal laser scanning microscopy showed decreased roughness in the series, [Bi_x_O_y_]^N^ > [Bi_x_O_y_-6% PVA]^N^ > [Bi_x_O_y_-C-6% PVA]^N^, and increased roughness for materials [Bi_x_O_y_]^S^, [Bi_x_O_y_-6% PVA]^S^, [Bi_x_O_y_-C-6% PVA]^S^, [Bi_x_O_y_]^P^, [Bi_x_O_y_-6% PVA]^P^ and [Bi_x_O_y_-C-6% PVA]^P^. The morphological analysis (electronic scanning microscopy) of the synthesized materials showed a wide variety of forms: overlapping nanoplates ([Bi_x_O_y_]^N^ or [Bi_x_O_y_]^S^), clusters of angular forms ([Bi_x_O_y_-6% PVA]^N^), pillars ([Bi_x_O_y_-6% PVA]^S^-Au), needle particles ([Bi_x_O_y_-Au], [Bi_x_O_y_-6% PVA]^S^-Au, [Bi_x_O_y_-C-6% PVA]^S^-Au), spherical particles ([Bi_x_O_y_-C-6% PVA]^P^-Pt), 2D plates ([Bi_x_O_y_]^P^-Pt) and 3D nanometric plates ([Bi_x_Oy-C-6% PVA]^S^-Au). For materials obtained in the first synthesis stage, antimicrobial activity increased in the series [Bi_x_O_y_]^N^ > [Bi_x_O_y_]^S^ > [Bi_x_O_y_]^P^. For materials synthesized in the second synthesis stage, when polymer (polyvinyl alcohol, PVA) was added, maximum antimicrobial activity, regardless of the microbial species tested, was present in the material [Bi_x_O_y_-6% PVA]^S^. For the materials synthesized in the third stage, to which graphite and 6% PVA were added, the best antimicrobial activity was in the material [Bi_x_O_y_-C-6% PVA]^P^. Materials synthesized and doped with metal ions (gold or platinum) showed significant antimicrobial activity for the tested microbial species.

## 1. Introduction

The continued excessive or incorrect use of antimicrobials in human and animal health accelerates the development of antimicrobial resistance. No class of antibiotics has been discovered since 1980; therefore, appropriate use of existing antimicrobial agents, along with the synthesis and development of new antimicrobial agents, is desirable [1,2].

In 2017, the World Health Organization (WHO) published the first list of priority pathogens which are resistant to antibiotics and, according to specialists, are the greatest threat to humanity. There is an urgent need of new antimicrobial agents against these bacteria. The following bacteria were classified as being of critical priority: *Acinetobacter baumannii, Pseudomonas aeruginosa* and *Enterobacteriaceae/Enterobacterales (Klebsiella pneumoniae*, *Escherichia coli*, *Enterobacter* spp., *Serratia* spp., *Proteus* spp., *Providencia* spp., *Morganella* spp.), *Enterococcus faecium* and *Staphylococcus aureus* [3].

The WHO subsequently developed a new list of pathogens that are at risk of triggering pandemics or serious bacterial infections and should be closely monitored [4]. Among these pathogens, Gram-negative bacteria are of particular importance because they show resistance to multiple antibiotics. This is due to the ability of these bacteria to find new ways to resist antibiotic treatment. Therefore, the list of priority pathogens has become a reference for the scientific community, indicating where efforts should be focused to manage the next epidemiological threat.

WHO strategies to reduce antimicrobial resistance aim to, among other goals, improve awareness and understanding of antimicrobial resistance [5,6], strengthen knowledge through surveillance and research [7], reduce the incidence of infection [8] and optimizing the use of antimicrobial agents [9].

For research to rapidly respond to these urgent public health needs, in recent years, numerous materials with antibacterial potential have been tested, especially against these bacterial strains. In the present work, several varieties of bismuth oxides are tested, which were shown to have significant antibacterial properties against both *P. aeruginosa* and *E. coli*, as well as *S. aureus*.

As part of the strategy to combat antibiotic resistance, EU Regulation 2019/6 establishes the limitation of the use of substances with antibiotic properties, both as therapy itself, but also in situations of use as adjuvants in various branches of industry, especially food [10].

Antimicrobial resistance is a complex process based on intrinsic bacterial features (the existing genetic material exhibits novel behaviours to avoid the action of the antibiotic), as well as acquired features (the bacterial adaptive capacity to gain new genetic material which gives the chance of bacterial survival despite antibiotic therapy). The list of resistance mechanisms within antibiotic classes are outlined by Darby et al. [11].

In many bacterial species (e.g., *Enterococcus* spp., *Staphylococcus* spp., *Streptococcus* spp., *Listeria* spp.), multi-antibiotic resistance is due to an interspecies transfer of resistance mediated by plasmids [12]. Over time, various metal nanoparticles, such as silver [13,14], copper [14,15], titanium [16,17,18,19], bismuth [15,20,21] and metal oxide particles, such as zinc oxide nanoparticles [15,22,23], titanium oxides [23,24,25] and bismuth oxides [26,27], have been studied to establish their efficacy in terms of antibacterial activity. The advantages of inorganic nanoparticles, in terms of antibacterial potential, include large surface-to-volume ratios, unique structural shapes and sizes of nanoparticles that allow interaction with bacteria. These characteristics of nanoparticles represent the main difference in interaction, when compared to microbial agents, allowing them to reduce the risk of antibacterial resistance. For example, the crystalline structure of bismuth oxide nanoparticles [26] can react with biological systems, including bacteria and fungi. One of the most important features of nanoparticles is that they reduce the probability to gain antimicrobial resistance, as they can be synthesized in a variety of shapes and sizes, which do not allow bacteria to adapt to these nanoparticles, and they have the ability to form free metal ions, which have a biocidal effect [9].

Bismuth oxides can be obtained by various methods: pulsed laser ablation in liquids [28,29,30,31], metal vapour oxidative deposition [32,33], sol-gel methods [34,35,36,37], wet chemical synthesis [38,39,40], solution combustion method [41,42], evaporation and thermal oxidation [43].

Bismuth oxide-based materials have a wide range of applications: in microelectronics [44,45,46,47,48], optical engineering [49], as a photo catalyst [50,51], surface disinfectant in hospitals and in the food industry [52], in pharmaceutical production [53], as an essential derivative in the manufacture of glass [54,55] and ceramics [56,57,58], in medicine [59,60] and as an agent in anticancer therapy [61].

The morphology and size of synthesized bismuth oxide nanoparticles have been found to be influenced by the production technique [30]. In this regard, in this paper, materials based on bismuth oxides have been synthesized, using the same synthesis technique, but modifying the acids used in synthesis (azotic, sulphuric, phosphoric) and it was found that the morphology of the synthesized particles is also different, with significant impact on the rate of inhibition of microbial growth.

Doping inorganic metal ion materials is a common option and can be used to recover these metal ions from used solutions, which later have the potential to ‘adapt’ and improve the properties of the materials on which they have been adsorbed, particularly to combat antimicrobial resistance [14,62,63]. Several studies have reported that the positive charge of the metal ion is responsible for antibacterial activity, allowing electrostatic attraction between the negative charge on the bacterial cell membrane and the positive charge of the nanoparticles [14,21].

The aim of the present work was to prepare three different series of bismuth oxide nanocomposites. In first series, desired material was prepared via sol–gel synthesis using bismuth carbonate as the precursor, and a white foam was obtained. Further, using the bismuth oxide foam obtained in first series, all nanocomposite materials from the second and third series were prepared, many of them presenting a gel structure.

## 2. Results and Discussion

### 2.1. The Characterisation of Bi_x_O_y_ Materials

Three series of materials have been prepared.

In Series I, materials were synthesized in the three acidic mediums (HNO_3_, H_3_PO_4_, H_2_SO_4_): [Bi_x_O_y_]^N/P/S^.

In Series II, 6% polymer PVA: [B_x_O_y_-6% PVA]^N/P/S^ was added to the materials from the first series, drop by drop.

In Series III, graphite (C) and 6% polymer (PVA) [Bi_x_O_y_-C-6% PVA]^N/P/S^ were added to materials from the first series.

**Figure 1:**
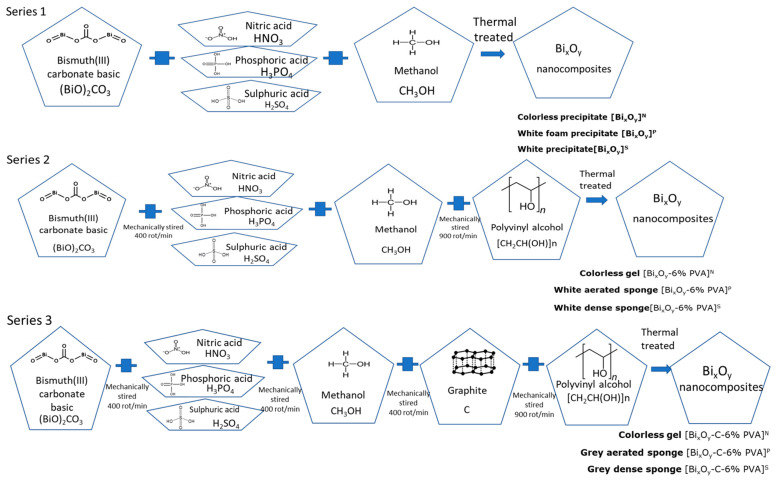
Schematic presentation of the synthesis of materials by the sol-gel method.

In Figure 1, the syntheses of materials are presented schematically.

The materials synthesized under different conditions were characterised by specific physicochemical methods in order to establish optimal synthesis conditions so as to present optimal antimicrobial activity.

#### 2.1.1. Thermogravimetric Analysis, TG

**Figure 2:**
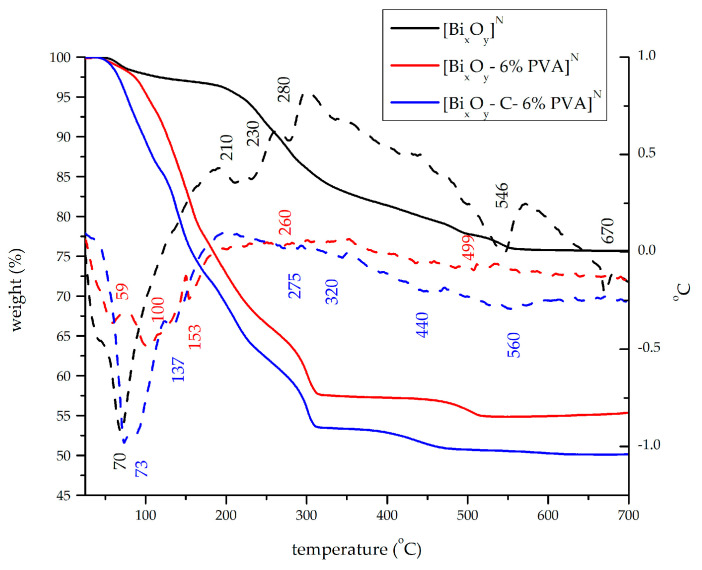
TG (solid line) and DTA (dashed line) for [Bi_x_O_y_]^N^, [Bi_x_O_y_-6% PVA]^N^ and [Bi_x_O_y_-C-6% PVA]^N^ systems.

**Figure 3:**
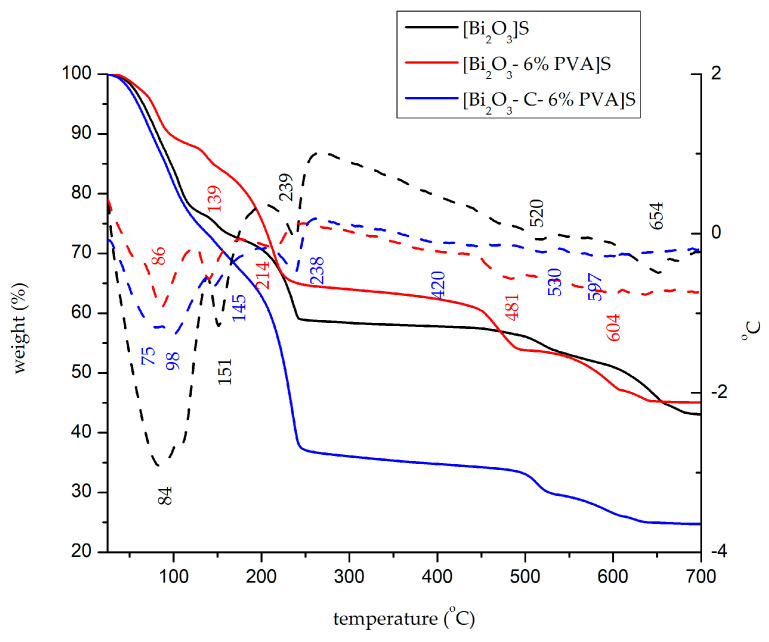
TG (solid line) and DTA (dashed line) for [Bi_x_O_y_]^S^, [Bi_x_O_y_-6% PVA]^S^ and [Bi_x_O_y_-C-6% PVA]^S^ system.

**Figure 4:**
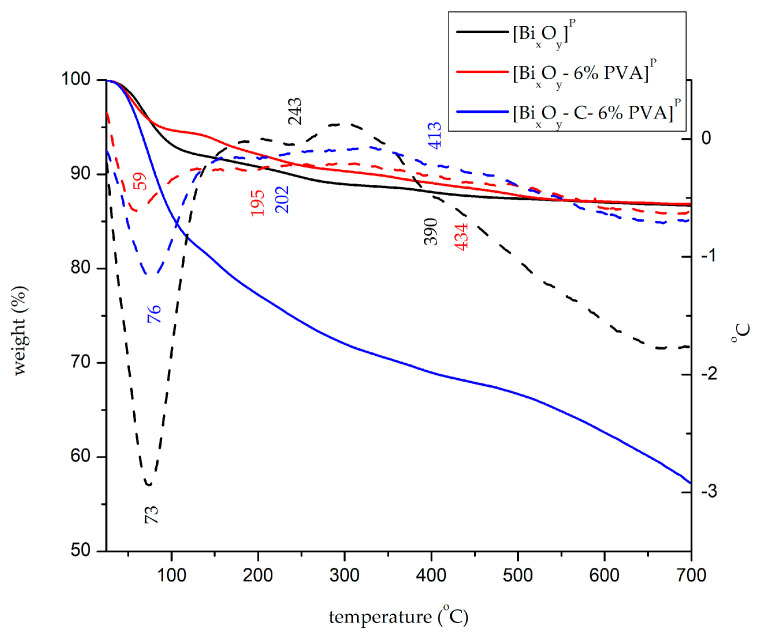
TG (solid line) and DTA (dashed line) for [Bi_x_O_y_]^P^, [Bi_x_O_y_-6% PVA]^P^ and [Bi_x_O_y_-C-6% PVA]^P^ systems.

Figure 2, Figure 3 and Figure 4 present the thermal decomposition curves for the three series of materials synthesized after the previous description, namely: [Bi_x_O_y_]^N/P/S^, [Bi_x_O_y_-6% PVA]^N/P/S^ and [Bi_x_O_y_-C-6% PVA]^N/P/S^, that were used to study the thermal stability of the materials.

Figure 2 depicts the thermal decomposition of the samples synthesized in the presence of nitric acid, and Table 1 shows the processes that take place for each system.

Following the analysis of thermal curves, shown in Figure 2, the four temperature zones where mass losses and specific thermal effects occurred were delimited. An overall analysis shows that for the sample [Bi_x_O_y_]^N^ there is a total loss weigh of 23.84%. With the addition of polivinylalchohol polymer (PVA), resulting in the material [Bi_x_O_y_-6% PVA]^N^, a total loss of mass of 45.14% is found in the system. When graphite is added, resulting in [Bi_x_O_y_-C-6% PVA]^N^, a total mass loss of 50.25% was determined.

For the sample [Bi_x_O_y_]^N^, the endothermic effects, presented in Table 1, accompanied by a weight loss of approximately 5%, were in the range of 25–200 °C (first endothermic effect). These effects are attributed to the removal of solvents and shallow adsorbtion of water. After the introduction of PVA in the material [Bi_x_O_y_-6% PVA]^N^ and graphite together with PVA in the material [Bi_x_O_y_-C-6% PVA]^N^, the mass losses were 22% and 28%, respectively. The 4- and 5-fold weight loss for these two materials may also be due to the presence of organic compounds added to the system (PVA and C).

The second endothermic effect occurred in the temperature range 200 to 400 °C, and results in a weight loss of approximately 14% for the [Bi_x_O_y_]^N^ sample and approximately 19% for the [Bi_x_O_y_-6% PVA]^N^ and [Bi_x_O_y_-C-6% PVA]^N^ samples. These were attributed to the decomposition of bismuth carbonate and the formation of β-Bi_2_O_3_. In the last temperature range of 400–700 °C, the weight loss of approximately 3% for all three samples is attributed to the total transformation of the material into α-Bi_2_O_3_ [64].

Figure 3 shows the thermal decomposition of the synthesized materials in the presence of sulphuric acid. Table 2 presents the processes that take place in the three cases.

Analysing the obtained data, we can see that when sulphuric acid was used in the synthesis of bismuth oxide, transformations were more complex than when nitric acid was used. Thus, the material [Bi_x_O_y_]^S^ has almost twice the total weight loss, 45.2%. Similar to the material synthesized in the HNO_3_ medium, the addition of PVA shows a greater mass loss (54.4% for [Bi_x_O_y_-6% PVA]^S^). In the case of material synthesized with graphite and PVA [Bi_x_O_y_-C-6% PVA]^S^, the mass loss is nearly doubled, approximately 75.6%. Transformations that take place on the TG curve in this system show four important endothermic processes. In the first part of the process, in the range 25–120 °C, for the sample [Bi_2_O_3_]^S^, a weight loss of approximately 13% is attributed to the loss of water and/or organic. In the case of the sample with the addition of PVA, [Bi_x_O_y_-6% PVA]^S^, no changes are observed within this temperature range; with a maximum of 86 °C, a mass loss of approximately 12% occured. In the case of [Bi_x_O_y_-C-6% PVA]^S^, the mass loss almost doubled, with a value of 26%, due to the graphite added. This occured in the same temperature range as above, and had two maximums, at 75 and 98 °C. This loss of mass can be attributed to partial decompositions of bismuth carbonate, acting as a catalyst.

In the second range of temperature, 120–175 °C, samples [Bi_x_O_y_]^S^, [Bi_x_O_y_-6% PVA]^S^ and [Bi_x_O_y_-C-6% PVA]^S^ show a weight loss of ~5%. Within this temperature range, mass loss is attributed to the decomposition of structural and partial water of bismuth carbonate. It is noted that by adding graphite and PVA, the transformations that take place in the system are faster.

The critical temperature range is the third range, between 175 and 300 °C, where the most important transformations take place. In this range, endothermic processes that occur around 230 °C are attributed to the loss of ${SO}_{4}^{2-}$ groups, the bismuth carbonate decomposition, and their changes to bismuth sulphate and bismuth oxide.

The last process that takes place between 350 and 700 °C shows endothermic effects with weight loss of ~13% being attributed to the consolidation of the structural and crystalline network of bismuth oxide and the total decomposition of polymers in the system.

Figure 4 shows the thermograms specific to the thermal decomposition of the samples synthesized in the presence of phosphoric acid. Table 3 shows the processes that take place in each system.

From the analysis of thermal curves performed for samples synthesized in the presence of phosphoric acid, it is noted that the sample [Bi_x_O_y_]^P^ indicates a total weight loss of 13.4%, similar to the sample to which PVA was added, [Bi_x_O_y_-6% PVA]^P^. In the case of the sample in which graphite and then PVA were added, the material [Bi_x_O_y_-C-6% PVA]^P^ had a total mass that was 3 times greater (42.7%). At the same time, it is noted that in each case, the materials undergo three important endothermic processes. In the first process that occurs between 25 and 120 °C, weight losses are observed specific to the loss of free water and organic solvents in the system. With the increase in temperature, in the second temperature range between 120 and 350 °C, an endothermic process is observed, with a maximum at approximately 200 °C, attributed to the decomposition of bismuth carbonate. Processes at this stage do not show significant losses because phosphate ions bind to bismuth ions, forming bismuth phosphate with a melting temperature greater than 400 °C. The addition of PVA to the system does not influence the total weight loss; it influences the structure of the compound, but does not form new bonds. The addition of graphite influences the system, affects the structure of the material and speeds up the process of obtaining bismuth oxide by acting as a catalyst.

Between 350 and 700 °C, most transformations take place. By inserting phosphate into the system, bismuth oxide begins to form after 400 °C, slowing down the process due to the addition of phosphate.

#### 2.1.2. X-ray Diffraction, DRX

**Figure 5:**
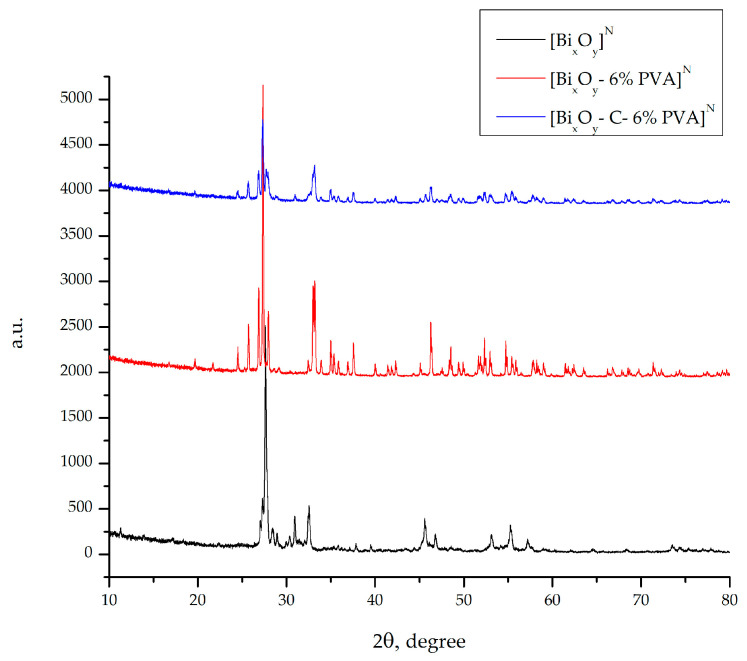
X-ray spectrum for [Bi_x_O_y_]^N^, [Bi_x_O_y_-6% PVA]^N^ and [Bi_x_O_y_-C-6% PVA]^N^ system.

**Figure 6:**
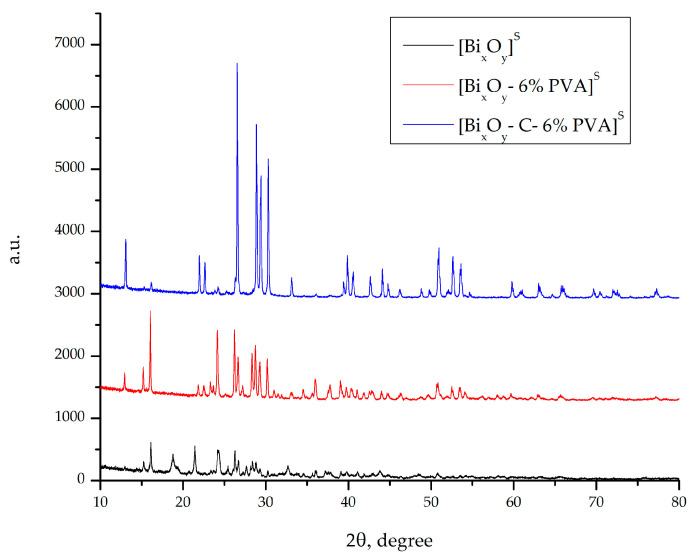
X-ray spectrum for [Bi_x_O_y_]^S^, [Bi_x_O_y_-% PVA]^S^ and [Bi_x_O_y_-C-6% PVA]^S^ system.

Figure 4, Figure 5 and Figure 6 show RX spectra for three series of materials synthesized in the three acidic media, with PVA 6% and with graphite and PVA 6% i.e., [Bi_x_O_y_]^N/P/S^, [Bi_x_O_y_-6% PVA]^N/P/S^ and [Bi_x_O_y_-C-6% PVA]^N/P/S^.

Thus, Figure 5 presents the difractograms of the three samples where nitric acid has been added to the reaction mass.

The samples [Bi_x_O_y_]^N^, [Bi_x_O_y_-6% PVA]^N^ and [Bi_x_O_y_-C-6% PVA]^N^ show similar difractograms in which a phase specific to the formation of bismuth oxide is identified, as well as additional peaks specific to the addition of graphite and PVA into the system. According to ICDD: 00-022-0515, a tetragonal crystallographic system of bismuth oxide is obtained as the main phase (Bi_2_O_3_) from the sample [Bi_x_O_y_]^N^. As a secondary phase, the material exhibits in a small proportion of bismuth oxide nitrate, ICDD: 00-051-525. The average crystalline size was calculated using the Scherrer Equation (1) [65],

(1) $$\tau =\frac{K\lambda }{\beta cos\theta }\;\;\;Scherrer\;equation$$

where τ is the mean size of the ordered (crystalline) domains, which may be smaller or equal to the grain size, and may be smaller or equal to the particle size; K is an adimensional shape factor, with a value close to unity, typically 0.9, but varies with the current shape of the crystallite; λ is the X-ray wavelength; β is the line broadening at half the maximum intensity (FWHM), after subtracting the instrumental line broadening, in radians; this quantity is also sometimes denoted as Δ(2θ) and θ is the Bragg angle. In this situation, a value of d = ~11 nm was calculated.

By adding PVA to the system, it was possible to purify the material by forming a single pure phase, that of bismuth oxide III. According to ICDD: 00-027-0053, material indexation [Bi_x_O_y_-6% PVA]^N^ are obtained as the single phase specific to bismuth III oxide with a monoclinical crystalline system, space group P2(1)/c,14 and network parameter a = 5.8480 [66]. The average size of the calculated crystals indicates that the material has a value of ~9 nm.

After the introduction of graphite into the system, the material undergoes changes in the structure, with its main phase as the crystalline form of bismutite, according to the ICCD: 00-025-1464. As a secondary phase, there is also a crystalline form of bismuth oxide, according to ICDD: 01-071-0465. A monoclinical crystalline system was observed, with space group P2(1)/c,14, but with a network parameter a = 5.8496, which causes the particle size to increase. For this phase, the crystalline system is a tetragonal system with the space group L4/mmm,139. The average size of the calculated crystals indicates that the material has a value of ~19 nm.

XRD spectra for the samples [Bi_x_O_y_]^S^, [Bi_x_O_y_-6% PVA]^S^ and [Bi_x_O_y_-C-6% PVA]^S^, in which sulphuric acid has been added to the system, are shown in Figure 6.

As the main crystalline phase, the material obtains the form of bismuth sulphate hydroxide, Bi(SO_4_)OH, according to ICDD: 04-025-4472, and bismuth phase sulphate oxides with crystalline structures are not defined, according to ICDD: 00-038-0507. At the same time, the sample difractogram [Bi_x_O_y_]^S^ shows the secondary peaks specific to the phase of monoclinical bismuth oxide C2/c E, according to ICDD: 00-050-0864. The average crystalline size of the material was determined using the Scherrer equation indicating a value of ~14 nm. Adding PVA to the system, in the sample [Bi_x_O_y_-6% PVA]^S^ causes changes in the structure, and improves the system. Thus, a crystalline phase of bismuth sulphate oxides is obtained that has two different structures and a secondary phase of bismuth oxide with a cubic crystalline system and Fm-3 m spatial group, according to ICDD: 00-016-0654. The first phase is given by bismuth sulphate oxides showing a monoclinical crystalline system with a C2/c,15 spatial group, with network parameters of =14.1659, according to ICDD: 01-088-7310. The second crystalline phase obtained, ICDD: 00-041-0686, indicates the formation of a bismuth sulphate oxide having a monoclinical crystalline system with a space group C2/c, 15 with network parameter a = 32.2150. The average size of crystals determined using Scherrer equation indicates a value of ~12 nm.

Adding graphite to the reaction mass, also changes the structure of the material by achieving 3 different phases. It is noted that the specific main phase is bismuth sulphate oxide, given by ICDD: 01-088-7310, which is also obtained in 6% PVA material. After adding graphite to the mass of the reaction, changes in the structure of the material are observed. The material indicates the formation of another crystalline structure of bismuth sulphate oxide, given by ICDD: 04-013-0544. As secondary phase, a bismuth oxide, given by ICDD is obtained: 01-071-0465, specific to a monoclinical crystalline system with space group P21/c, 14, and carbon initially introduced into the system. The calculation of the size of the crystals was performed using the Scherrer equation, indicating a value of 9 nm.

**Figure 7:**
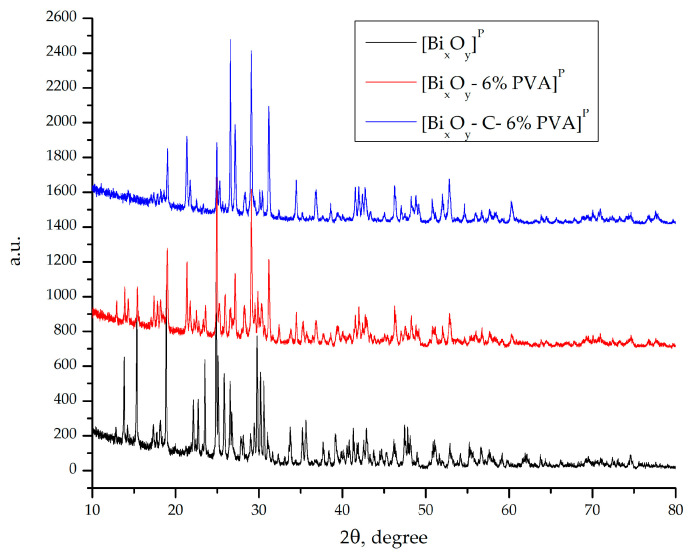
X-ray spectrum for [Bi_x_O_y_]^P^, [Bi_x_O_y_-6%PVA]^P^ and [Bi_xO_-C-6%PVA]^P^ system.

XRD spectra for samples [Bi_x_O_y_]^P^,[Bi_x_O_y_-6% PVA]^P^ and [Bi_x_O_y_-C-6% PVA]^P^ to which phosphoric acid has been added to the system are shown in Figure 7.

The diffraction spectrum of the sample [Bi_x_O_y_]^P^ indicates that two main crystalline phases were obtained with the addition to the system of phosphoric acid. After evaluating the sample, according to ICDD: 01-083-2755, a crystalline phase of bismuth phosphate (Bi(PO_4_)) was obtained with a monoclinical P2_1_/m spatial group. The second crystalline phase obtained as indicated by the peaks of the crystalline system of bismuth phosphate (Bi_2_P_4_O_13_) with the monoclinical group C2/c, observed in ICDD: 00-043-0638. As the secondary phase of the system, according to ICDD: 00-050-0864, a crystalline phase of monoclinical bismuth oxide C2/c E was obtained. The mean crystalline size was calculated using the Scherrer equation, achieving a value of d = 10 nm.

By adding PVA, small changes in the crystalline structure are observed. The main crystalline phase given by the system is indicated by the bismuth phosphate (Bi(PO_3_)_3_) monoclinical P2_1_/c, according to ICDD: 00-043-0471, and changes in the crystalline structure are observed, obtaining bismuth phosphate (Bi(PO_4_)). As the secondary phase, the tetragonal bismuth oxide P-4b2 is observed, according to ICDD: 00-018-0244. The average crystalline size was calculated using the Scherrer equation, achieving a value of d = 12 nm.

After adding graphite to the system, the same crystalline phases are observed for the material [Bi_x_O_y_-6% PVA]^P^, but this time, the main phase is the crystalline phase of BiPO_4_. At the same time, the specific phase of graphite is indicated by ICDD: 00-056-0159. The size of the crystals, determined by the Scherrer equation, was d~9 nm.

#### 2.1.3. Fourier Transform Infrared Spectroscopy, FT-IR

**Figure 8:**
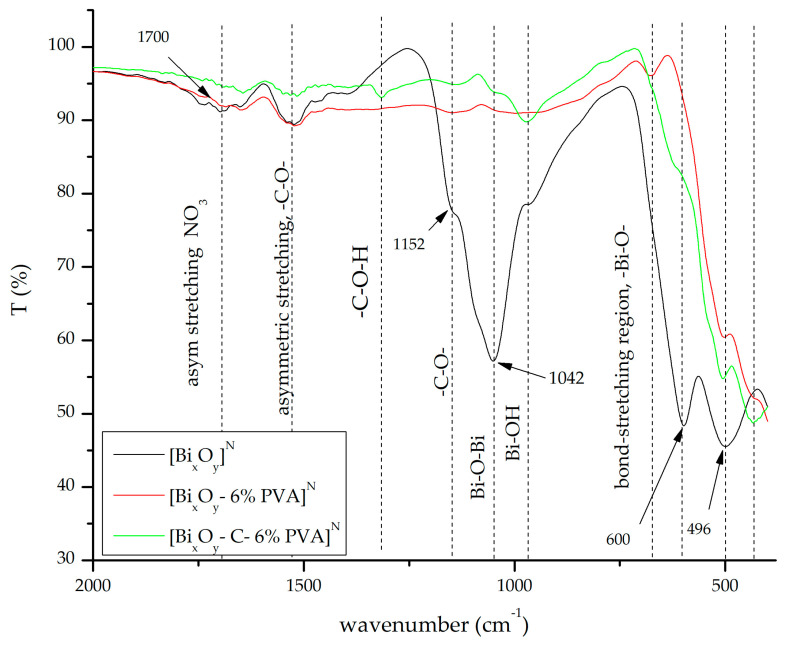
FT-IR spectrum for [Bi_x_O_y_]^N^, [Bi_x_O_y_-6% PVA]^N^ and [Bi_x_O_y_-C-6% PVA]^N^ materials.

**Figure 9:**
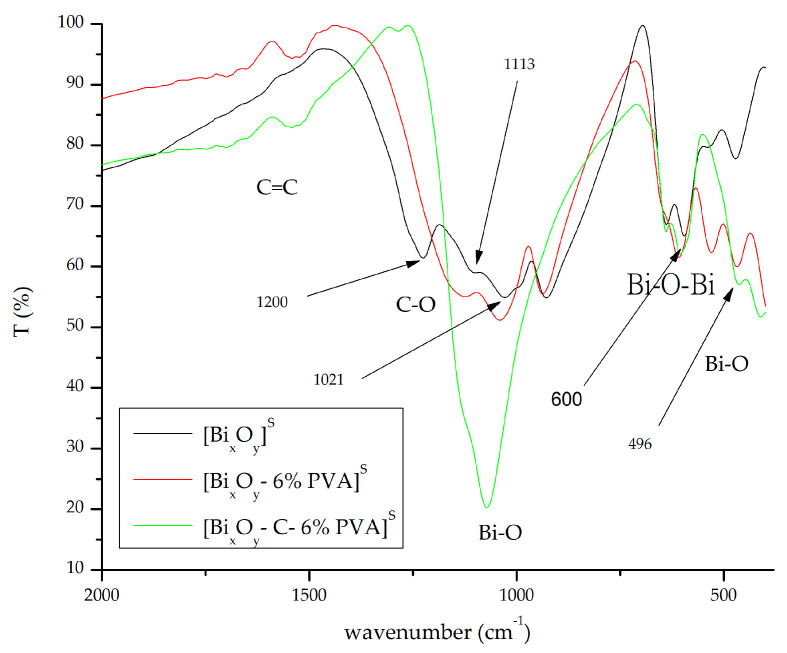
FT-IR spectrum for [Bi_x_O_y_]^S^, [Bi_x_O_y_-6% PVA]^S^ and [Bi_x_O_y_-C-6% PVA]^S^ materials.

**Figure 10:**
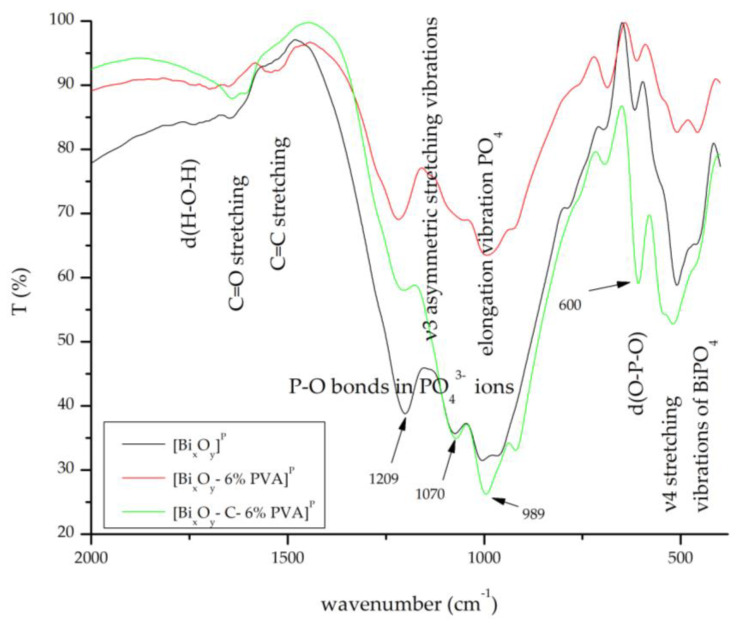
FT-IR spectrum for [Bi_x_O_y_]^P^, [Bi_x_O_y_-6% PVA]^P^ and [Bi_x_O_y_-C-6% PVA]^P^ materials.

FT-IR spectra for the three series of synthesized materials, [Bi_x_O_y_]^N/P/S^, [Bi_x_O_y_-6% PVA]^N/P/S^ and [Bi_x_O-C-6% PVA]^N/P/S^ are shown in Figure 8, Figure 9 and Figure 10.

The FT-IR spectrum for the sample in which nitric acid was introduced is shown in Figure 8.

Compared with XRD spectra, it was observed that when PVA and graphite were added to the system, major changes in structure occur. In the case of basic samples, [Bi_x_O_y_]^N/S^, the tetragonal crystalline phase of Bi_2_O_3_ was obtained, confirmed by XRD. In the case of the sample [Bi_x_O_y_]^P^, the specific crystalline phase for α-Bi_2_O_3_ was found. From the FT-IR spectrum (Figure 8) the presence of bismuth oxide specific strips from ~496, 600, 964, 1046 and 1152 cm^−1^ was observed [64,67,68]. The confirmation that Bi_2_O_3_ has been obtained is given by the presence of the wave number specific vibration ~1042 cm^−1^ which is specific to the Bi-O bond [69]. The presence of vibrations at wave number ~1700 cm^−1^ indicates that the material still has ${NO}_{3}^{-}$ groups in the system. At the same time, the presence of C-O-vibration from ~1500 cm^−1^ and 1250 cm^−1^ indicates that excess methanol in the system restores carbonate’s bonds and does not fully break down. After the addition of PVA polymer, the system has a batochrome shift to smaller wave numbers. At the same time, it is observed that by adding the polymer PVA, the specific phase of the crystallographic system α is formed, highlighted by the presence of vibrations in the wave number 440 cm^−1^ [70].

In the sample where sulphuric acid was added, the spectrum shown in Figure 9 shows the formation of specific strips for bismuth sulphate oxides. The specific bands of the compound are found under the wave numbers: 1200 cm^−1^, 1113 cm^−1^, 1021 cm^−1^ and 925 cm^−1^. The vibration from 1113 cm^−1^ is specific to the ${SO}_{4}^{2-}$ groups [71]. The secondary phase of bismuth oxide is observed by the presence of strips from 496 cm^−1^, 600 cm^−1^ and 1040 cm^−1^.

When sulphuric acid has been replaced by phosphoric acid (Figure 10), several changes occur in the system. When correlated with Rx spectrum data (Figure 7), it is noted that the main specific phase is given by bismuth phosphate. By following Figure 9, the vibrations specific to the band specific for bismuth phosphate (ν3 asymmetric stretching) can be observed, at wave numbers: 1209 cm^−1^, 1070 cm^−1^ and 989 cm^−1^. There are also specific strips for bismuth phosphate (ν4 asymmetric stretching) from 600 cm^−1^, 510 cm^−1^ and 425 cm^−1^ [72].

#### 2.1.4. Scanning Electron Microscopy, SEM

**Figure 11:**
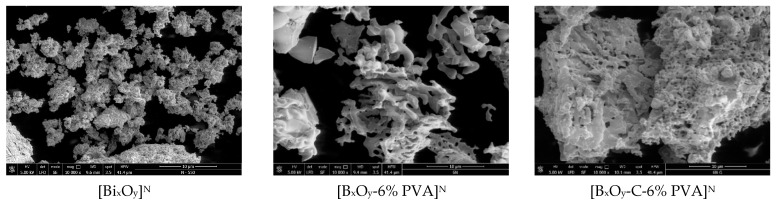
SEM for [Bi_x_O_y_]^N^, [Bi_x_O_y_-6%PVA]^N^ and [Bi_x_O_y_-C-6%PVA]^N^ materials.

**Figure 12:**
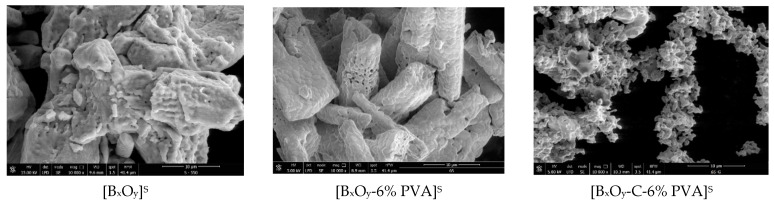
SEM for [Bi_x_O_y_]^S^, [Bi_x_O_y_-6%PVA]^S^ and [Bi_x_O_y_-C-6%PVA]^S^ materials.

**Figure 13:**
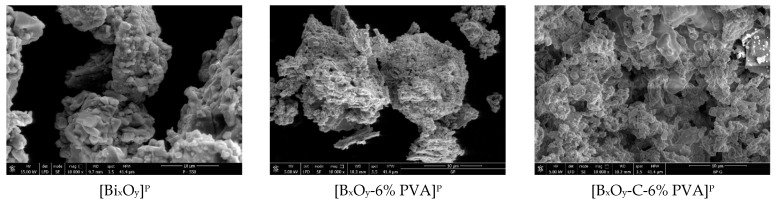
SEM for [Bi_x_O_y_]^P^, [Bi_x_O_y_-6% PVA]^P^ and [Bi_x_O_y_-C-6% PVA]^P^ materials.

SEM micrographs for the three series of synthesized materials, [Bi_x_O_y_]^N/P/S^, [Bi_x_O_y_-6% PVA]^N/P/S^ and [Bi_x_O_y_-C-6% PVA]^N/P/S^, are shown in Figure 11, Figure 12 and Figure 13.

The morphologies of materials that have been synthesized with nitric acid are shown in Figure 11.

Analysing the morphology of the material [Bi_x_O_y_]^N^, it is observed that it has overlying nanoplates that form aggregates of approximately 1–2 µm. It is also noted that their distribution is uniform, probably due to heat treatment at 550 °C. After adding the PVA, the materials [Bi_x_O_y_-6% PVA]^N^ exhibit an increase in size to 10 µm, forming clusters, and the particles show an angular shape. When graphite and PVA are added, in the material [Bi_x_O_y_-C-6% PVA]^N^, the addition of graphite influences the formation of large clusters, above 30 µm in size. The materials have a semi-rusty surface.

The morphologies of samples in which sulphuric acid was used for synthesis are shown in Figure 12.

When sulphuric acid was used for the synthesis, major changes occurred in the system. In the case of the base material [Bi_x_O_y_]^S^, clusters of overlying nanoplates, greater than 50 µm, are formed. Interestingly, when the PVA polymer is introduced into the system [Bi_x_O_y_-6% PVA]^S^, major changes occur in its structure, forming particles with micrometric dimensions and a much more orderly distribution. In this context, the polymer plays a role in separating particles by depositing them around a cluster and maintaining the shape of the particle. By adding graphite to the material [Bi_x_O_y_-C-6% PVA]^S^, it can be seen that the particles disperse into smaller aggregates forming chains connected with each other, but with an undefined structure.

Figure 13 shows the materials to which phosphoric acid has been added to the system.

By analysing the SEM images in Figure 13, the materials tend to form plates with nanometric dimensions. In the case of base material [Bi_x_O_y_]^P^, the shapes are random; however, with the addition of polymer and graphite and polymer, the materials [Bi_x_O_y_-6% PVA] and [Bi_x_O_y_-C-6% PVA]^P^, respectively, tend to form microplates. However, [Bi_x_O_y_-C-6% PVA]^P^ has a much different appearance of compared to the other samples, exhibiting greater homogeneity over the entire analysed surface, without many spacings between the formed agglomerations.

#### 2.1.5. Confocal Laser Scanning Microscopy, CLSM

Data on the roughness of the three series of synthesized materials, [Bi_x_O_y_]^N/P/S^, [Bi_x_O_y_-6% PVA]^N/P/S^ and [Bi_x_O_y_-C-6% PVA]^N/P/S^, are shown in Table 4; images recorded and used for all these calculations are presented in Figure 1 of the supplementary material.

The alignment of the Sq and SA data shown in Table 4 to the SEM images (Figure 11, Figure 12 and Figure 13) can confirm the previous statements. Thus, in the case of nitric acid synthesis materials [Bi_x_O_y_]^N^ and those obtained after the addition of PVA [Bi_x_O_y_-6% PVA]^N^ and graphite and polymer [Bi_x_O_y_-C-6% PVA]^N^ show a decrease in roughness. When sulphuric acid is used, the roughness of the materials increases with the addition of polymer, [Bi_x_O_y_-6% PVA]^S^, and graphite and polymer, [Bi_x_O_y_-C-6% PVA]^S^. A particular situation can be observed in the case of the phosphorus sample, [Bi_x_O_y_-C-6% PVA]^P^, which, after the addition of the graphite and PVA, shows a 5-fold increase in roughness compared to the rest of the samples. The different trend of the sample [Bi_x_O_y_-C-6% PVA]^P^ occurs due to its more compact and homogeneous appearance compared to the other samples, as demonstrated by the SEM images (Figure 13). There is a correlation between the roughness data obtained using CLSM and the data obtained for the specific surface determined with the BET method (Table 5), that is supported by SEM images (Figure 11, Figure 12 and Figure 13). This is observed for all samples, except [Bi_x_O_y_-C-6% PVA]^P^, which has a tendency to form clusters or agglomerations that influence the roughness data obtained. Clusters tend to be chaotic or intercalated; thus, the maximum layer height (Sp) and the maximum layer depth (Sv) cannot be precisely determined. Therefore, different data were obtained from the specific surfaces (Table 5). It should also be noted that the roughness data obtained with the BET method comprise the surface of the entire sample; i.e., the data were not influenced by the way in which the layers are formed. The specific surface is influenced by several aspects such as porosity, pore size, pore distribution, particle shape and size and roughness, which together determine the minimisation or maximisation of the specific surface [64]. Except for the [Bi_x_O_y_-C-6% PVA]^P^ sample, which has a more homogeneous appearance, most samples have spongy surfaces, are separated into clusters and have different morphologies and roughness. For samples [Bi_x_O_y_]^S^, morphology is in the form of plates, and for the sample [Bi_x_O_y_-6% PVA]^S^, morphology indicates the passing of plates in the pillars, while the material [Bi_x_O_y_-C-6% PVA]^S^ has the form of small pile chains, which causes increased roughness. The cause of the increase in roughness is due to the intercalation, agglomeration and chaotic settlement of layers and particles as in the case of [Bi_x_O_y_-C-6%PVA]^P^.

#### 2.1.6. Brunauer–Emmett–Teller (BET) Surface Area Analysis

Measurements of the specific surface of the materials were made (Table 5) and the specific area in the specific range 0.05–0.3 P/P_o_ was determined for samples [Bi_x_O_y_]^N/P/S^, [Bi_x_O_y_-6% PVA]^N/P/S^ and [Bi_x_O_y_-C-6% PVA]^N/P/S^.

Analysing specific surface data and correlating the data with SEM images (Figure 11, Figure 12 and Figure 13) and roughness data (Table 4), it can be concluded that the main processes take place on the surface of the material. Adding graphite and polymer to the system changes the structure of the material, by blocking the pores, thus decreasing the specific surface, observed in Table 5. The largest surfaces were obtained from the synthesis of materials with nitric acid.

#### 2.1.7. Doping of Materials with Metal Ions, Au(III) and Pt(IV)

To highlight the presence of the metal ion (Au(III) and Pt(IV) respectively) on the surface of the three series of synthesized materials, [Bi_x_O_y_]^N/P/S^, [Bi_x_O_y_-6% PVA]^N/P/S^ and [Bi_x_O_y_-C-6% PVA]^N/P/S^, the materials were characterised by SEM, EDX (see Figure S1) and CLSM. The specific surface area of metal ion-doped materials was also determined.

#### 2.1.8. Scanning Electron Microscopy, SEM and Energy Dispersive X-ray Analysis, EDX after Doping

**Figure 14:**
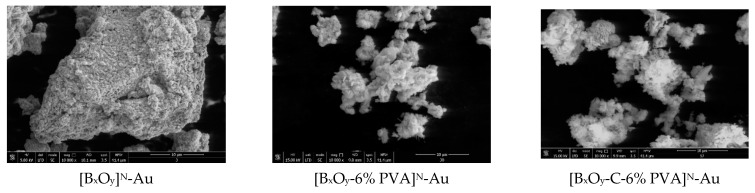

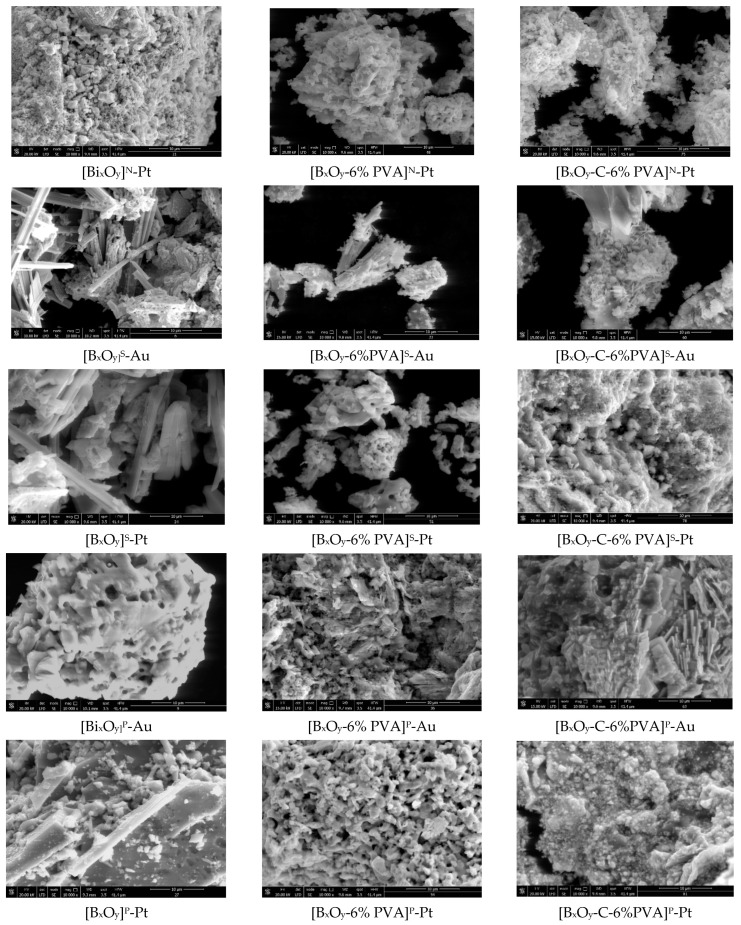
SEM for materials [Bi_x_O_y_]^N/P/S^, [Bi_x_O_y_-6% PVA]^N/P/S^ and [Bi_x_O_y_-C-6% PVA]^N/P/S^ doped with gold and platinum.

In Figure 14, the morphologies of the materials synthesized and doped with the ions of Au(III) and Pt(IV) are shown.

By doping with Au(III), changes in the morphology of the materials can be observed, namely agglomeration or dispersion of particles, captured in SEM images (Figure 14). For samples synthesized with nitric acid, not doped with metal ions, the tendency is to form spongy material and large clusters; in the presence of Au(III) ions, the clusters shrink with the addition of polymer, PVA and graphite and PVA. When adding graphite and PVA and doping with Au (III), the material [Bi_x_O_y_-C-6% PVA]^N^-Au appears as 2D plates on the surface, with nanometric dimensions, stratified.

The second category of materials, those synthesized in the presence of sulphuric acid and then doped with Au(III), exhibit pile-shaped particles, similar to the samples without Au(III), but much thinner ([Bi_x_O_y_]^S^-Au) and with needle agglomerations ([Bi_x_O_y_-6% PVA]^S^-Au) or crystals on their surface ([Bi_x_O_y_-C-6% PVA]^S^-Au). Cumulatively, it can be found that the presence of Au(III) on the surface of the synthesized materials with H_2_SO_4_ changes the structure of the material on the surface, thus, explaining the increase in the specific surface area observed after BET analysis.

For the category of materials synthesized in the presence of phosphoric acid and doped with Au(III), it is noted that the presence of Au(III) ions causes the formation of large, compact clusters, which become more homogeneous when adding PVA ([Bi_x_O_y_-6% PVA]^P^-Au). It has the appearance of large, 2D, agglomerated plates when graphite and PVA ([Bi_x_O_y_-C-6% PVA]^P^-Au) are added.

The material synthesized with nitric acid, doped with Pt (IV), ([Bi_x_O_y_]^N^-Pt), is homogenized and compacted, compared to the undoped base material ([Bi_x_O_y_]^N^); this trend disappears when graphite and PVA are added. When graphite alone was added to the system, clusters in the form of smaller agglomerations were observed.

With the addition of Pt(IV) ions in samples with sulphuric acid, their structure changes, similar to the samples doped with Au (III) ions, but with larger dimensions of the pillar ([Bi_x_O_y_]^S^-Pt). However, the addition of Pt(IV) to the material with PVA ([Bi_x_O_y_-6% PVA]^S^-Pt) and to the graphite and PVA material ([Bi_x_O_y_-C-6% PVA]^S^-Pt) changes the structure, the pillars disappear and no longer form nanoplates, but a homogeneous compound cluster forms.

The basic sample synthesized with phosphoric acid, [BixOy] P, is very different in the presence of Pt(IV) ions, with 2D plates with micrometric dimensions, which overlap each other. However, this aspect changes significantly when polymer is added, the material [Bi_x_O_y_-6% PVA]^P^-Pt forms a sponge-like structure with an interconnected structure. Once graphite has been added to the system, the material [Bi_x_O_y_-C-6% PVA]^P^-Pt again changes its structure by forming nanometre-sized spherical particles that are linked one with another, forming large clusters. Therefore, we can specify that the material [Bi_x_O_y_-6% PVA]^P^-Pt is more homogeneous than the material [Bi_x_O_y_-C-6% PVA]^P^.

In general, it can be specified that platinum ions have an influence on the appearance of the sample, the final impact being the modification of the structure of the surface of the materials.

#### 2.1.9. Confocal Laser Scanning Microscopy, CLSM, after Doping

The roughness of the materials synthesized in the three series: [Bi_x_O_y_]^N/P/S^, [Bi_x_O_y_-6% PVA]^N/P/S^ and [Bi_x_O_y_-C-6% PVA]^N/P/S^ and subsequently doped with Au(III) and Pt(IV) is shown in Table 6.

The roughness of the materials changes due to the change in the shape of the samples, a fact that is confirmed by the SEM analysis. Thus, in the case of Au(III) ions, the material with the highest roughness is [Bi_x_O_y_-C-6% PVA]^S^-Au, which has a more prominent 3D shape compared to the rest of the samples. Even among 3D-shaped samples, the material [Bi_2_O_3_-C-6% PVA]^S^-Au has the highest roughness value. However, the relationship between the results obtained for the specific surface and the roughness of the synthesized materials is not only attributed to changes in morphology resulting from the addition of PVA and graphite and PVA. It is also based on the visible change in particle size in the SEM images. This trend was also observed by Rahimi et al. who showed that surface roughness decreases when particle size decreases, while the specific surface area tends to increase [65]. At the same time, it should be noted that the placement of the layers, i.e., distributed in a chaotic or orderly manner, as well as the morphology of the surface have an important influence on the final results of the roughness and the specific surface area.

In the case of materials synthesized and doped with Pt (IV), the material with the highest roughness value is [Bi_x_O_y_-C-6%PVA]^P^-Pt, which had the highest Sz values (as a result of the sum Sp and Sv, which are also high compared to the other samples; Table 6). Changes in roughness data after adding PVA and graphite together with PVA are based on the same causes outlined above; however, it is not possible to confirm which of them (morphology, intercalation of layers, or particle size) are major contributors or their proportional contributions. In addition to those mentioned, metal ions also enter a combination, Au(III) and Pt(IV), having primary effects on the structure of the material [66]. At the same time, the amount of metal that will enter into the existing structure or adhere to the surface of the material depends on the porosity of the material and also on the shape of the pores, thus allowing a larger or smaller amount of metal ions to form bonds with the material.

#### 2.1.10. Brunauer–Emmett–Teller, BET, Surface Area Analysis after Doping

The specific surface areas of the Au(III) and Pt(IV) doped materials are presented in Table 7.

It is noted that in the case of materials in which Au(III) was introduced, the surface area is significantly greater, compared to those to which Pt(IV) was added. The largest specific area obtained for these materials was for the material [Bi_2_O_3_-C-6%PVA]^S^–Au, which is also observed from the analyses performed by SEM (Figure 14) and CLSM (Table 6). The smallest specific area is observed for material [Bi_x_O_y_-C-6% PVA]^P^-Pt.

#### 2.1.11. Antibacterial Performance

**Figure 15:**
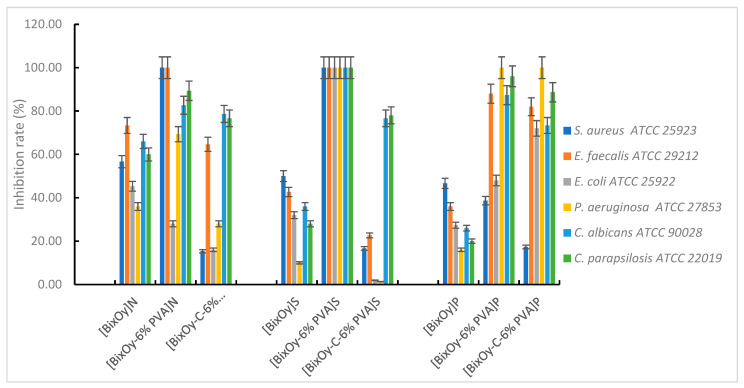
Antimicrobial activity of materials [Bi_x_O_y_]^N/P/S^, [Bi_x_O_y_-6% PVA]^N/P/S^, [Bi_x_O_y_-C-6% PVA]^N/P/S^.

The microbiological tests carried out showed that the synthesized materials showed antimicrobial activity, which varied depending on the type of material used and the microbial species tested (Figure 15).

With regard to materials [Bi_x_O_y_]^N^, [Bi_x_O_y_-6% PVA]^N^ and [Bi_x_O_y_-C-6% PVA]^N^, the antimicrobial activity was influenced by the addition of PVA and the addition of graphite and PVA, respectively.

In the first step, with the addition of polymer, the antimicrobial capacity of Bi_2_O_3_ synthesized with HNO_3_ is improved, regardless of the microbial species tested, except for *E. coli*. In Gram-positive bacteria, total bactericidal activity is observed, as proven by the 100% inhibition rate of bacterial growth for *S. aureus* and *E. faecalis*. In contrast, with the addition of graphite to the system, antimicrobial activity is significantly reduced. One possible explanation would be that the specific surface area decreases in series [Bi_x_O_y_]^N^ > [Bi_x_O_y_-6% PVA]^N^ > [Bi_x_O_y_-C-6% PVA]^N^ and the particle size increases from 11 nm for [Bi_x_O_y_]^N^ to 19 nm for [Bi_x_O_y_-C-6% PVA]^N^, highlighting the importance of the particle size ratio to the contact surface when interacting with the surface of bacterial cells.

For materials synthesized with H_2_SO_4_, the addition of polymer to the system significantly improved antimicrobial activity, when, regardless of the species tested, the microbial growth inhibition rate was 100%. By adding graphite to the system, the material [Bi_x_O_y_-C-6% PVA]^S^ disperses into small needle aggregates, forming chains with an undefined structure. When PVA is introduced into the system, major changes occur in the structure of the material [Bi_x_O_y_-6% PVA]^S^, forming particles in the form of piles of micrometric dimensions, ordered. Even if the specific surface area decreases in series [Bi_x_O_y_]^S^ > [Bi_x_O_y_-6%PVA]^S^ > [Bi_x_O_y_-C-6% PVA]^S^, the trend of increasing the roughness of the materials from [Bi_x_O_y_]^S^ to [Bi_x_O_y_-C-6% PVA]^S^ has been observed, in which case, the particle morphology (pillars, acicular shapes) and roughness appear to have played a definitive role in the appearance of the antimicrobial effect. As mentioned, the data on the roughness of the samples include analysis on the surface of the entire sample, not influenced by the way in which the component layers are placed, while the specific surface is influenced by several aspects such as porosity, pore size, pore distribution, shape and size of particles and roughness. Thus, the adhesion of the test material on the surface of the microbial cells was facilitated, which subsequently led to disturbance of the permeability of the microbial membrane, with a final effect on cell death.

Materials based on bismuth oxides synthesized with H_3_PO_4_ have a different morphological shape from the rest of the samples, showing greater homogeneity throughout the analysed area, in the form of microplates, without much spacing between the formed agglomerations. Compact morphology does not allow for high adhesion on the surface of microbial cells, which is reflected in a weaker interaction with the bacterial cell, manifested by a decrease in the bacterial growth inhibition rate by materials [Bi_x_O_y_]^P^ compared to materials [Bi_x_O_y_]^N^, regardless of the tested microbial species.

By comparison, metal ion-doped and synthesized materials exhibited significant antimicrobial activity.

**Figure 16:**
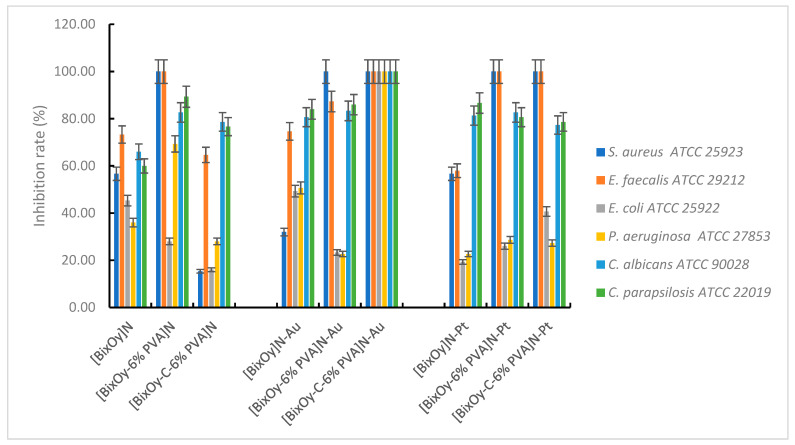
Antimicrobial activity of materials with HNO_3_.

Materials [Bi_x_O_y_]^N^, [Bi_x_O_y_-6% PVA]^N^ and [Bi_x_O_y_-C-6% PVA]^N^ synthesized using HNO_3_ and doped with metal ions showed antimicrobial activity as shown in Figure 16.

For samples synthesized with HNO_3_ and doped with Au (III), the tendency to form a spongy texture is observed. This facilitates the adsorption of materials, resulting in good adhesion with the microbial cell and their penetration into cells. Consequently, an increase in the microbial inhibition rate is observed, especially for materials of type [Bi_x_O_y_-C-6% PVA]^N^-Au, at which the microbial growth inhibition rate was 100%.

It is possible that the structure of the material [Bi_x_O_y_-C-6% PVA]^N^-Au, in which Bi-O bonds remain, through the addition of graphite to the system, would allow the existence of free spaces, leading to increased oxidation activity. Oxidation activity is also enhanced by the presence of Au(III), which causes an overall increased oxidative activity leading to a material showing antimicrobial efficacy against *S. aureus, E. faecalis, E. coli, P. aeruginosa, C. albicans* and *C. parapsilosis*. The total bactericidal effect was also observed upon *S. aureus* and *E. faecalis* in materials [Bi_x_O_y_-6% PVA]^N^-Pt and [Bi_x_O_y_-C-6% PVA]^N^-Pt, possibly also facilitated by the presence of blank spaces in the structure of the material with a high oxidation potential.

The antibacterial mechanism in this case may be attributed to a combined effect between the chelation effect of Au(III) or Pt(IV) metal ions from the material structure (which causes perturbation in the membrane function of the microbial cell) and the oxidation effect generated by reactive oxygen species (ROS). Generation of ROS-type anionic radicals, induced by the presence of metal ions, has the effect of destroying highly oxidative cell components, such as unsaturated fatty acids or phospholipid residues on the surface of the cell membrane [10]. The cell wall of Gram-positive bacteria has a thick layer of peptidoglycan containing amino acids, lipids and surface proteins, all of which have the role of barrier and protection of the cell, which is not easily crossed by ROS. As a result, negatively charged ROS cannot infiltrate into the Gram-positive bacterial cytomembrane and remain on the surface of the bacterial cell, resulting in an attack on highly oxidative cell components, causing its destruction over time.

In Gram-negative bacteria such as *E. coli* and *P. aeruginosa*, the cell wall still has an external membrane containing lipopolysaccharides, porins and a thin layer of peptidoglycan. Therefore, the stronger activity of the synthesized materials against Gram-positive bacteria, compared to Gram-negative bacteria, is attributed to the difference in the formation of the cell walls. In the case of Gram-negative bacteria, their cell walls make it difficult to attack ROS generated by the oxides resulting from synthesis.

However, consideration should be given to the morphology of the surface of the synthesized materials, which has been observed to vary significantly, both after the first stage of synthesis (by using different acids in which the precursor is dissolved) and after addition to the system of the polymer and graphite with polymer. This can be the starting point in the synthesis of materials with specific antimicrobial properties directed toward a particular pathogen, considering the specificity of the microbial cell membrane and the morphology of the material with antimicrobial properties.

Considering that in the case of synthesis involving H_2_SO_4_ and H_3_PO_4_, other oxides (BiOSO_4_, Bi_2_PO_4_) were also obtained intermediately; it is possible to obtain multilayer particles of bismuth oxides. Their surface was altered and their nanometric dimensions facilitate interaction with the microbial cell membrane and result in specific cytotoxicity observed in the case of materials [Bi_x_O_y_-C-6% PVA]^N^-Au.

**Figure 17:**
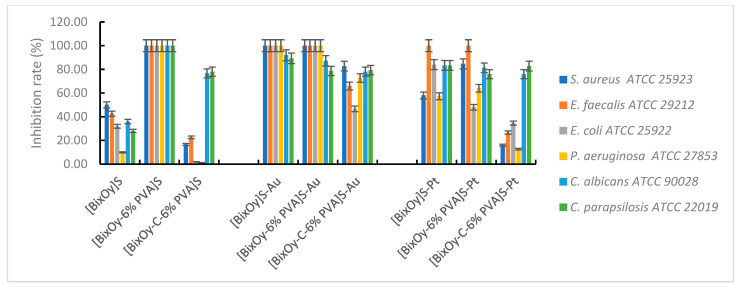
Antimicrobial activity of materials with H_2_SO_4_.

Regarding the second category of materials, those synthesized in the presence of sulphuric acid and then doped with metal ions, Au(III) and Pt (IV), the manifestation of the antimicrobial effect, translated into the inhibition rate of microbial growth, is shown in Figure 17.

The materials [Bi_x_O_y_]^S^-Au, [Bi_x_O_y_-6% PVA]^S^-Au and [Bi_x_O_y_-C-6% PVA]^S^-Au exhibit thin pile-shaped particles with needle growth, while the materials [Bi_x_O_y_-6% PVA]^S^-Pt and [Bi_x_O_y_-C-6% PVA]^S^-Pt have the shape of larger pillars with a tendency to homogeneity.

Data from the characterisation of materials synthesized in the presence of sulphuric acid and then doped with Au(III) showed the nanometric size and the acicular structure of the particles, which allowed good adsorption of these materials anddisruption of nutrient exchange, which subsequently leads to cell destruction. The maximum bacterial growth inhibition rate is reached for both Gram-positive and Gram-negative bacteria tested. By doping with Pt(IV), the materials [Bi_x_O_y_-6% PVA]^S^-Pt and [Bi_x_O_y_-C-6% PVA]^S^-Pt have a much more homogeneous aspect, which gives the materials a smaller area for binding to the bacterial cell wall. Implicitly, as the exchange of nutrients in microbial cells takes place more easily and the cells survive longer, the rate of inhibition of bacterial growth being much lower.

**Figure 18:**
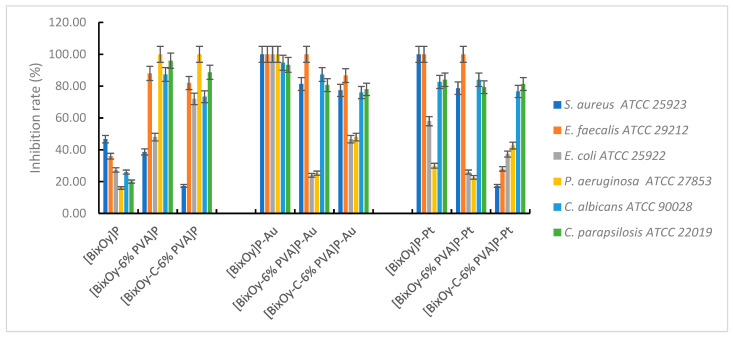
Antimicrobial activity of materials with H_3_PO_4_.

The expression of the antimicrobial effect of materials synthesized using H_3_PO_4_ is summarised in Figure 18.

Au(III) doping of these materials results in a maximum inhibition of bacterial growth for *S*. *aureus, E. faecalis, E. coli* and *P. aeruginosa*. It seems that the decisive role is played by Au(III), through its electronegativity and chelation capacity, in contact with the surface of bacterial cells. With the addition of PVA to the system, materials of the type [Bi_x_O_y_-6% PVA]^P^-Au exhibit a reduced bactericidal effect, especially in the case of Gram-negative bacteria. The behaviour is similar in the case of materials [Bi_x_O_y_-6% PVA]^P^-Pt, with a higher bacterial growth inhibition rate for *S. aureus* and *E. faecalis*, compared to *E. coli* and *P. aerugiosa*.

Gram-negative bacteria have a lipopolysaccharide layer on the outside, and these negative charges on the lipopolysaccharides are attracted to the weak positive charge of the nanoparticles. On the other hand, the cell wall of Gram-positive bacteria is composed mainly of a thick layer of peptidoglycan consisting of linear polysaccharide chains cross-linked through short peptides to form a three-dimensional rigid structure. The rigidity and extended reticular system not only reduce the places for anchoring of nanoparticles on the bacterial cell wall, but they make the bacterial wall itself more difficult to penetrate.

It is possible that the oxidising potential of formed oxides is also diminished by the compact morphology of synthesized materials. These do not allow the external bacterial membrane to cross, and the ROS attack is difficult to achieve or cell lysis takes a longer time. This assumption is also supported by the effect of the material [Bi_x_O_y_-C-6%PVA]^P^-Au. Although it was proved to have the largest specific area, the tendency of the particles to agglomerate did not allow for a uniform distribution. Thus, the material does not easily adhere to the surface of cell membranes, which delays the antimicrobial effect of the material.

However, it is possible that there is an extremely important bactericidal role for the crystalline structure of the materials synthesized with HNO_3_, when producing α-Bi_2_O_3_. Moreover, with the use of H_2_SO_4_ or H_3_PO_4_, in addition to Bi_2_O_3_, bismuth oxidic sulphates (BiOSO_4_) and bismuth phosphates (BiPO_4_) are also obtained, which decrease the oxidative potential of the materials.

## 3. Materials and Methods

### 3.1. The Preparation of Bi_x_O_y_ Materials

#### Summary of Basic Materials [Bi_x_O_y_]^N/P/S^

In a Berzelius beaker, 5 g of bismuth precursor, bismuth carbonate (BiO)_2_CO_3_ is placed, and 10 mL acid was added (HNO_3_, H_3_PO_4_, H_2_SO_4_) to dissolve carbonate (precursor: acid molar ratio = 1:2). They were mixed with a magnetic stirrer at 400 rot/min.

In the first series of samples, the nature of the acid varied. (I) With the addition of HNO_3_ 65%, the reaction that occurs is exothermic and, at the end, after homogenization for 30 min, the material becomes colourless. Then, 50 mL of methanol was added and homogenized for 30 min, forming at the end a colourless precipitate, [Bi_x_O_y_]^N^. (II) After the addition of H_3_PO_4_ 85%, following all the previous steps, the material becomes a white foam. Then, 50 mL of methanol was added and homogenized for 30 min, finally forming a white foam precipitate [Bi_x_O_y_]^P^. (III) After the addition of H_2_SO_4_ 96%, maintaining the previous steps, the material becomes a white paste. Then, 50 mL of methanol was added and homogenized for 30 min, finally leading at a white precipitate formation, [Bi_x_O_y_]^S^.

The second series was obtained by introducing, drop by drop, 6% PVA into the system, on the base material [Bi_x_O_y_]^N/P/S^ and shaking obtained mixture for 5 min at 900 rot/min. The material [Bi_x_O_y_]^N^ (I) obtained in Series I becomes a colourless gel, [Bi_x_O_y_-6% PVA]^N^). The material obtained (II) in Series I was a white foam, and after adding the polymer, a white aerated sponge is formed ([Bi_x_O_y_-6% PVA]^P^). When using H_2_SO_4_ 96%, the (III) result was a white paste, and after adding the polymer, a dense white sponge is formed ([Bi_x_O_y_-6% PVA]^S^).

Series III was obtained from the base materials to which 0.1 g of graphite (C) was added and the polymer PVA 6% was later added because the reaction mass immediately precipitated. For (I), the material remains colourless gel ([B_x_O_y_-C-6% PVA]^N^). The material in the form of an aerated white sponge (II) turns grey ([B_x_O_y_-C-6% PVA]^P^). The material in the form of a dense white sponge for the sample [Bi_x_O_y_-6% PVA]^S^ (III) turns grey after the addition of the graphite and the polymer [Bi_x_O_y_-C-6% PVA]^S^.

All materials obtained in the three series were separated by vacuum filtration. Separate precipitations were dried in the oven, approximately 24 h, up to constant temperature and then calcined in the oven in air atmosphere at 550 °C for 4 h.

### 3.2. The Characterisation of Bi_x_O_y_ Materials

#### 3.2.1. Thermogravimetric Analysis, TG

The thermal analysis was performed using a Mettler thermo-analyser system TGA/SDTA 851/LF/1100 (Mettler-Toledo LLC, Columbus, OH, USA). The sample with mass of approximately 10 mg was placed in aluminum crucibles of 150 μL. The experiments were carried out in air atmosphere with a heating rate of 10 °C/min.

#### 3.2.2. X-ray Diffraction, XRD

The Powder X-ray Diffraction measurements were performed using a Rigaku Ultima IV (Tokyo, Japan) instrument with a sample to detector distance of 185 mm, using the Cu_Kα_ radiation (λ = 1.5418 Å). The diffraction patterns were recorded with 5°/min, in the 2θ interval between 10 and 80° (70° oscillation angle), with 0.01 steps (exposure time = 0.12 s).

#### 3.2.3. Scanning Electron Microscopy, SEM and Energy Dispersive X-ray Analysis, EDX

To highlight the morphology of the synthesized materials, SEM micrographs were performed, in a low vacuum system, using LFD detector to prevent shadowing effect of the particles. For the SEM analysis of the synthesized materials, the samples were fixed on aluminium stubs. The accelerating voltage used for the sample’s irradiation was between 5–20 kV, with a spot size of 3.5 and a pressure of 50 Pa. The free working distance (FWD) was around 10 mm. To determine the elemental composition of the synthesized materials, EDX spectroscopy was performed. Both SEM micrographs and EDX spectroscopies were performed using the EIF Quanta EGF 250 instrument (FEI, Hillsbro, OR, USA).

#### 3.2.4. Fourier Transform Infrared Spectroscopy, FT-IR

To highlight the groups specific to each synthesized material, infrared spectroscopy with the Fourier transform, FT-IR, was used. All FT-IR spectra were recorded on solid samples by using an IRAffiniy-1S SHIMADZU spectrophotometer (Shimadzu Corporation, Kyoto, Japan) equipped with attenuated reflection module (ATR module). In order to have a minimum error all the spectra were recorded in triplicate into the range 4000 to 400 cm^−1^, with a resolution of 0.5 cm^−1^. Peak and band identification were made by using peak detection command embedded into the spectrophotometer specific software (LabSolutions IR).

#### 3.2.5. Confocal Laser Scanning Microscopy, CLSM

Confocal laser scanning microscopy (CLSM) is an optical imaging technique for increasing optical resolution and contrast of a microscope by means of using a spatial pinhole to block out-of-focus light in image formation. The images of the synthesized materials were taken using the OLYMPUS OLS 4000 confocal microscope laser. Determination of synthesized material roughness was carried out by 2D and 3D laser scanning. The scanned surface showed 1279 × 1280 μm, and the magnification used was ×10^8^. Using a specific formula, some parameters designate the texture or appearance of the analyzed samples. In all experiments, a quantity of 0.5 g of material was used, which was fixed on aluminum stabs.

#### 3.2.6. Brunauer–Emmett–Teller (BET) Surface Area Analysis

The specific surface area was determined using the Brunauer–Emmett–Teller (BET) method using Quantachrome Nova 1200E equipment. The samples were degassed into the vacuum at room temperature for 24 h. The analyses were carried out by using a 0.1 g sample at a temperature of 77 K in the nitrogen atmosphere.

#### 3.2.7. Doping of Materials with Metal Ions, Au(III) and Pt(IV)

In order to improve the antimicrobial activity of materials, all samples were doped with metal ions (at concentration of 50 mg/L) known for their antimicrobial properties, namely Au(III) and Pt(IV) [73]. Doping was carried out in such a way that the metal ion:material ratio (*v*/*w*) is 10:1, at pH < 3, according to previous studies by the authors [74,75]. The samples were left in contact for 24 h at room temperature, then dried in the oven (Pol-eko model SLW 53, SDT, Wodzisław Śląski, Poland) for 24 h at 323 K.

#### 3.2.8. Antibacterial Performance

The synthesized materials were tested for antimicrobial activity. For this, preliminary microbiological tests were performed using heterotrophic bacterial inoculum and subsequently we confirmed the antimicrobial activity using reference microbial strains belonging to the American Type Culture Collection (ATCC).

Both the basic materials, after each synthesis step, i.e., [Bi_x_O_y_]^N/P/S^, [Bi_x_O_y_-6% PVA]^N/P/S^ and [Bi_x_O_y_-C-6% PVA]^N/P/S^, and base materials doped with metal ions i.e., [Bi_x_O_y_]^N/P/S^-Au, [Bi_x_O_y_-6% PVA]^N/P/S^-Au and [Bi_x_O_y_-C-6% PVA]^N/P/S^-Au and [Bi_x_O_y_]^N/P/S^-Pt, [Bi_x_O_y_-6% PVA]^N/P/S^-Pt and [Bi_x_O_y_-C-6% PVA]^N/P/S^-Pt were tested to highlight their antimicrobial properties.

For each heterotrophic bacterial inoculation, tryptic soy agar gel (VWR Chemicals, Belgium) was used for the isolation, counting, and culturing of microorganisms. The culture medium has the following composition: pancreatic digest of casein 15 g/L, papaic digest of soy bean 5 g/L, sodium chloride 5 g/L and agar 15 g/L. The medium was prepared according to the manufacturer’s instructions and sterilised, then poured into Petri plates.

Bacterial inoculum was adjusted for a final concentration of 0.5 McFarland (1–2 × 10^8^ CFU/mL). The solid material to be tested was weighed using 0.2 g for each test. Microbiological tests were performed with 3 repetitions to ensure the accuracy of the results. Petri plates after inoculation were incubated under specific conditions for 48 h at 310 K. After incubation, Petri plates were read with an automatic bacterial colony counter (YUL Instruments, Barcelona, Spain) to determine the number of bacterial colonies, which was subsequently used to estimate the antibacterial effect of the tested material.

To confirm the antimicrobial effect, after conducting preliminary microbiological tests using heterotrophic bacterial inoculum, the materials tested on reference microbial strains were selected. The effect on Gram-positive bacteria was followed on *Staphylococcus aureus* and *Enterococcus faecalis*, Gram-negative bacteria used were *Escherichia coli* and *Pseudomonas aeruginosa*, and fungal strains used were *Candida albicans* and *Candida parapsilosis* (Thermo Scientific, Waltham, MA, USA). Using the same nutrient medium, tryptic soy agar (VWR Chemicals, Leuven, Belgium), the samples were incubated 48 h at 310 K, and then read with the automatic bacterial colony counter (Flash&Go, YUL Instruments, Barcelona, Spain).

The microbial growth inhibition rate was then calculated using the formula (Equation (2)):

(2) $$inhibition\;rate=\frac{\left[{UFC}_{control}-{UFC}_{test}\right]}{{UFC}_{control}}\times 100$$

where *UFC_control_* = the number of colonies on the control plate, and

*UFC_test_* = the number of colonies on the flat test.

Total antimicrobial activity was considered when the rate of inhibition of microbial growth was 100%.

## 4. Conclusions

According to the performed microbiological tests, it was found that the synthesized materials showed antimicrobial activity, which varied depending on the type of used material and the tested microbial species. For materials obtained in the first synthesis stage, antimicrobial activity increased in series [Bi_x_O_y_]^N^ > [Bi_x_O_y_]^S^ > [Bi_x_O_y_]^P^.

For the synthesized materials to which polymer was added, the maximum antimicrobial activity, regardless of the microbial species tested, was with [Bi_x_O_y_-6% PVA]^S^. For the synthesized materials to which graphite and polymer were added, the best antimicrobial activity was with [Bi_x_O_y_-C-6% PVA]^P^.

The morphology of bismuth oxide particles is a key point in the design of biologically active materials. Although the same precursor was used to obtain these materials in inorganic synthesis, the different dissolution media had different influences on the morphology of the particles. Understanding the kinetics of dissolving bismuth carbonate into various acids, associated with the presence of specific inorganic ligands that result in bismuth oxides with different morphologies, is crucial to determine their antimicrobial activity. Among the bismuth species, spherical and spongy forms are the most common morphologies, but cylindrical forms, pillars, or needle shapes were also obtained in this study. Their specific interactions with microbial cells facilitated the manifestation of the antimicrobial effect.

In some cases, the introduction of PVA or graphite and PVA in the reaction mass decreased the ability of the material to induce oxidative stress, since there was a reduction in the bacterial inhibition rate, for example, in the case of the effect of materials [Bi_x_O_y_]^N^, [Bi_x_O_y_-6% PVA]^N^ and [Bi_x_O_y_-C-6% PVA]^N^ on *E. coli*.

Doping of bismuth oxide-based materials with Au(III) and Pt(IV) generally increased the cytotoxicity of nanoparticles, especially in the case of Au(III)-doped materials. The antibacterial effect appears to be conferred by the electronegativity of metal ions, the nanometric size of the materials and the increased surface, through which they destroy the membrane, cross the microbial cell wall, and create intracellular lesions. The structural difference in the cell walls of Gram-positive and Gram-negative bacteria the materials obtained has a significantly smaller effect on the growth of Gram-positive bacteria.

## Figures and Tables

**Table 1:** The thermal processes that take place for [Bi_x_O_y_]^N^, [Bi_x_O_y_-6% PVA]^N^ and [Bi_x_O_y_-C-6% PVA]^N^ systems.

Material	Lost Weight (%)	Temperature Range (°C)	Temperature Process (°C)
[Bi_x_O_y_]^N^	5.50	25–200	70
	14.3	200–450	210, 230 and 280
	3.46	450–600	546
	0.54	600–700	670
[B_x_O_y_-6% PVA]^N^	5.85	25–120	59 and100
	16.9	120–175	153
	19.7	175–350	260
	2.56	350–700	499
[B_x_O_y_-C-6% PVA]^N^	14.1	25–120	73
	13.8	120–175	137
	19.1	175–350	275 and 320
	3.18	350–700	440 and 560

**Table 2:** The thermal processes for [Bi_x_O_y_]^S^, [Bi_x_O_y_-6% PVA]^S^ and [Bi_x_O_y_-C-6% PVA]^S^.

Material	Lost Weight (%)	Temperature Range (°C)	Temperature Process (°C)
[B_x_O_y_]^S^	13.5	25–120	84
	4.08	120–175	151
	13.6	175–350	239
	13.8	350–700	520 and 654
[B_x_O_y_-6% PVA]^S^	12.0	25–120	86
	4.27	120–175	139
	19.1	175–350	214
	19.1	350–700	481 and 604
[B_x_O_y_-C-6% PVA]^S^	26.4	25–120	75 and 98
	6.72	120–175	145
	31.0	175–350	238
	11.4	350–700	450, 530 and 597

**Table 3:** The thermal processes for [Bi_x_O_y_]^P^, [Bi_x_O_y_-6% PVA]^P^ and [Bi_x_O_y_-C-6% PVA]^P^ systems.

Material	Lost Weight (%)	Temperature Range (°C)	Temperature Process (°C)
[Bi_x_O_y_]^P^	8.56	25–175	73
	2.72	175–350	243
	2.12	350–700	390
[B_x_O_y_-6% PVA]^P^	5.56	25–120	57
	2.33	120–350	195
	5.28	350–700	434
[B_x_O_y_-C-6% PVA]^P^	17.9	25–120	76
	5.35	120–350	202
	19.3	350–700	413

**Table 4:** Roughness of samples.

Sample	Sq (µm)	SSK	SKU	SP (µm)	SV (µm)	SZ (µm)	SA (µm)	MType	λc (µm)
[Bi_x_O_y_]^N^	11.4	−0.31	1.83	25.164	20.7	45.9	10.1	R	80
Bi_x_O_y_-6% PVA]^N^	10.7	−0.22	3.13	39.047	31.8	70.8	8.61	R	80
[B_x_O_y_-C-6% PVA]^N^	8.63	0.32	4.09	34.773	26.0	60.7	6.24	R	80
[B_x_O_y_]^S^	4.58	−1.30	5.75	21.398	12.8	34.2	3.16	R	80
B_x_O_y_-6% PVA]^S^	9.08	−0.86	2.96	29.673	19.4	49.2	7.46	R	80
[B_x_O_y_-C-6% PVA]^S^	10.7	−0.02	1.79	23.697	21.9	45.6	9.45	R	80
[Bi_x_O_y_]^P^	20.0	−0.07	4.62	94.809	94.1	188.9	13.4	R	80
Bi_x_O_y_-6% PVA]^P^	9.08	−0.86	2.96	29.6	19.4	49.2	7.46	R	80
[B_x_O_y_-C-6% PVA]^P^	48.8	−0.30	5.88	246.8	176.4	423.3	31.1	R	80

**Table 5:** The specific surface area of the materials, determined by the BET method.

Sample	Surface Area (m^2^/g)
[Bi_x_O_y_]^N^	3.11
[B_x_O_y_-6% PVA]^N^	2.06
[B_x_O_y_-C-6% PVA]^N^	0.71
[B_x_O_y_]^S^	2.01
[B_x_O_y_-6% PVA]^S^	1.25
[B_x_O_y_-C-6% PVA]^S^	0.07
[Bi_x_O_y_]^P^	1.12
[B_x_O_y_-6% PVA]^P^	0.77
[B_x_O_y_-C-6% PVA]^P^	0.02

**Table 6:** Confocal laser scanning microscopy for [Bi_x_O_y_]^N/P/S^, [Bi_x_O_y_-6% PVA]^N/P/S^ and [Bi_x_O_y_-C-6% PVA]^N/P/S^ doped with Au(III) and Pt(IV).

Sample	Sq (µm)	SSK	SKU	SP (µm)	SV (µm)	SZ (µm)	SA (µm)	MType	λc (µm)
Au(III)									
[Bi_x_O_y_]^N^	8.50	−2.06	8.88	18.7	48.4	67.2	6.36	R	80
[Bi_x_O_y_]^P^	6.30	−0.73	2.60	32.6	45.1	77.7	5.07	R	80
[B_x_O_y_]^S^	6.35	−0.25	2.83	18.1	17.0	35.1	5.11	R	80
Bi_x_O_y_-6% PVA]^N^	7.64	−1.18	4.29	12.5	31.4	44.0	6.24	R	80
Bi_x_O_y_-6% PVA]^P^	6.06	0.14	4.47	23.7	19.6	43.4	4.46	R	80
B_x_O_y_-6% PVA]^S^	7.80	−0.43	1.86	10.8	18.0	28.8	6.86	R	80
[B_x_O_y_-C-6%PVA ^N^	9.92	−0.91	3.25	23.1	34.1	57.2	8.03	R	80
[B_x_O_y_-C-6%PVA]^P^	6.17	−0.99	3.33	11.1	19.6	30.8	4.70	R	80
[B_x_O_y_-C-6%PVA]^S^	16.9	−1.11	3.85	16.7	28.8	45.6	15.34	R	80
Pt(IV)									
[Bi_x_O_y_]^N^	8.04	−1.40	4.59	15.1	35.2	50.4	6.02	R	80
[Bi_x_O_y_]^P^	6.50	−0.44	2.36	11.8	23.0	34.8	5.54	R	80
[B_x_O_y_]^S^	4.13	0.58	5.17	18.8	16.1	35.0	2.92	R	80
Bi_x_O_y_-6% PVA]^N^	7.19	−0.74	2.43	10.8	19.6	30.5	6.11	R	80
Bi_x_O_y_-6% PVA]^P^	7.27	−1.14	5.15	20.6	37.3	58.01	5.10	R	80
B_x_O_y_-6% PVA]^S^	6.60	−1.21	4.88	15.5	26.7	42.24	4.67	R	80
[B_x_O_y_-C-6%PVA]^N^	6.50	−0.44	2.36	11.8	23.0	34.85	5.54	R	80
[B_x_O_y_-C-6%PVA]^P^	9.64	−1.25	5.45	26.2	46.3	72.5	6.86	R	80
[B_x_O_y_-C-6%PVA]^S^	8.08	−0.45	2.10	12.2	20.7	33.0	6.76	R	80

**Table 7:** Surface area for [Bi_2_O_3_-C-6% PVA]^N/P/S^ materials doped with Au(III) and Pt(IV).

Sample	Surface Area (m^2^/g)
[Bi_2_O_3_-C-6% PVA]^N^-Au	2.37
[Bi_2_O_3_-C-6% PVA]^P^-Au	7.80
[Bi_2_O_3_-C-6% PVA]^S^-Au	20.5
[Bi_2_O_3_-C-6% PVA]^N^-Pt	4.23
[Bi_2_O_3_-C-6% PVA]^P^-Pt	0.39
[Bi_2_O_3_-C-6% PVA]^S^-Pt	3.50

## Data Availability

The data that support the findings of this study are available from the corresponding author upon reasonable request.

## Supplementary Materials

The following supporting information can be downloaded at: https://www.mdpi.com/article/10.3390/ijms241713173/s1.
