# Supplementary material for: Synthesis, Characterization and Antimicrobial Activity of Multiple Morphologies of Gold/Platinum Doped Bismuth Oxide Nanostructures
Source: Int J Mol Sci. 2023 Aug 24;24(17):13173. doi: 10.3390/ijms241713173 (PMC10488132; doi:10.3390/ijms241713173)
Supplement: Supplementary file 1 [file ijms-24-13173-s001.zip › ijms-2536828-supplementary.pdf]

Materials	2D CLSM	3D CLSM
$[Bi_2O_3]^N$	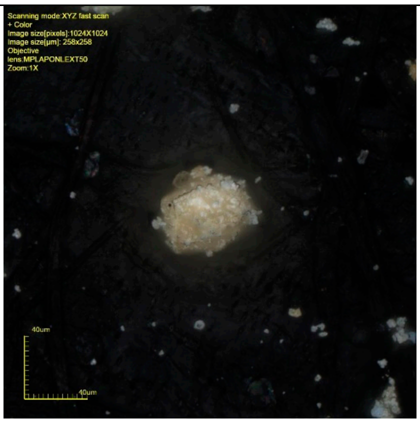	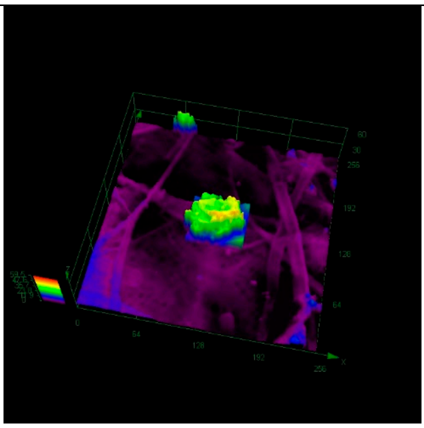
$[Bi_2O_3]^P$	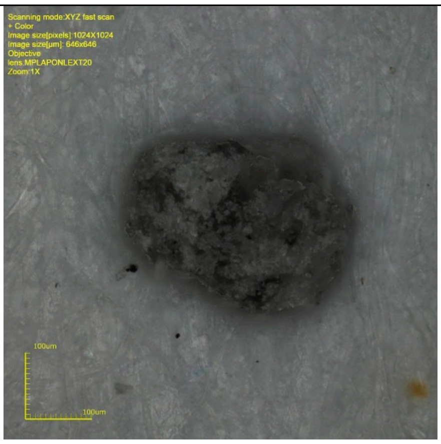	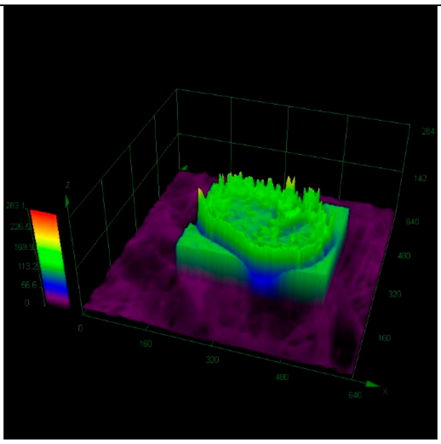
$[Bi_2O_3]^S$	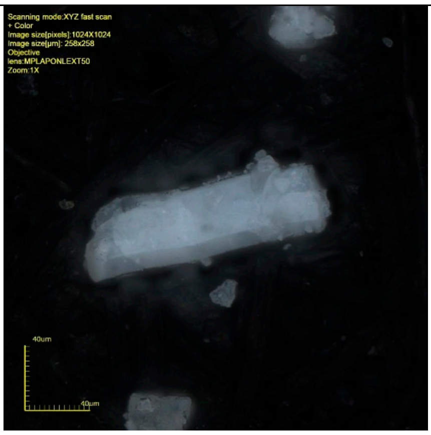	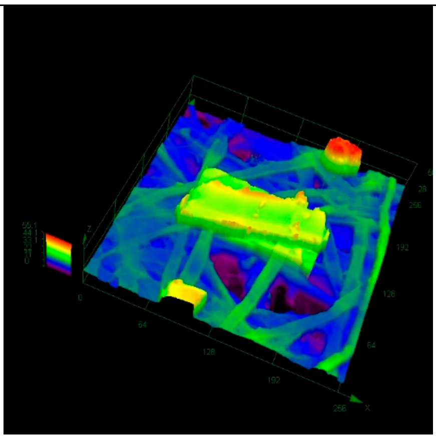

<table>
<tr><td>[Bi_2O_3- 6% PVA]^N</td><td>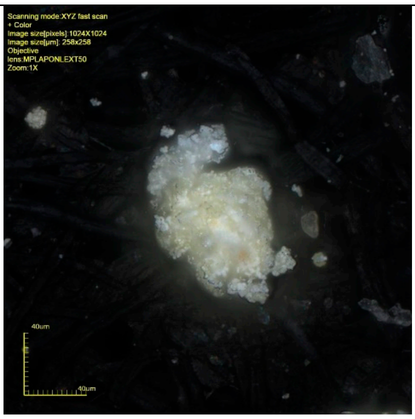
</td><td>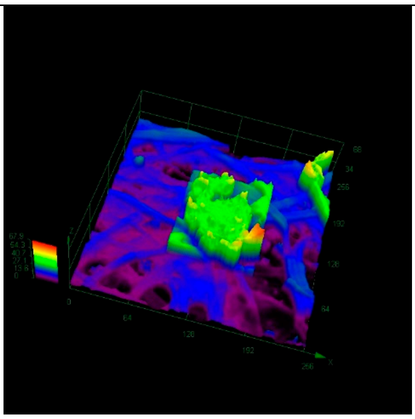</td></tr>
<tr><td>[Bi_2O_3- 6% PVA]^P</td><td>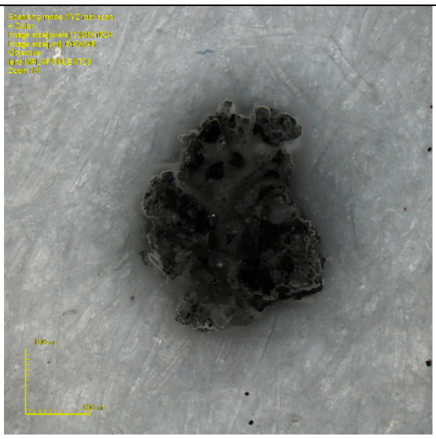</td><td>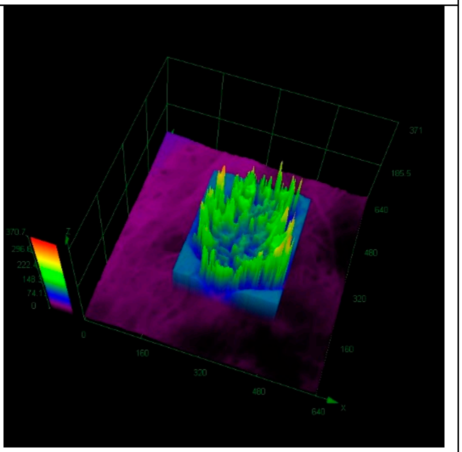</td></tr>
<tr><td>[Bi_2O_3- 6% PVA]^S</td><td>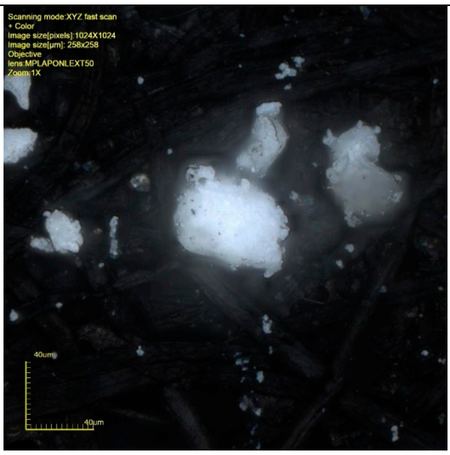
</td><td>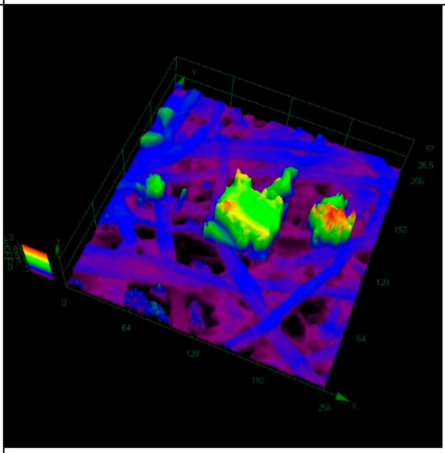</td></tr>
</table>

[Bi_2O_3- C-6% PVA][N]	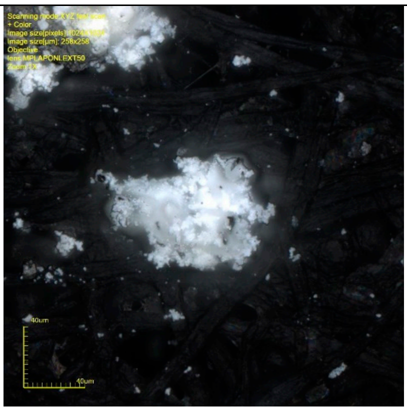	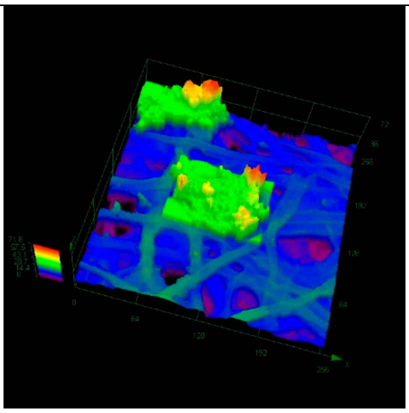
[Bi_2O_3- C-6% PVA][P]	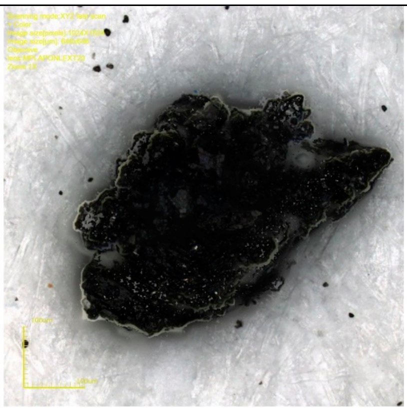	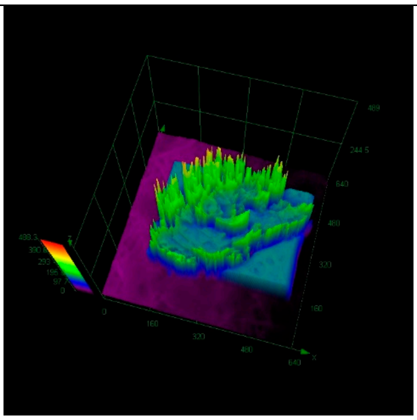
[Bi_2O_3- C-6% PVA][S]	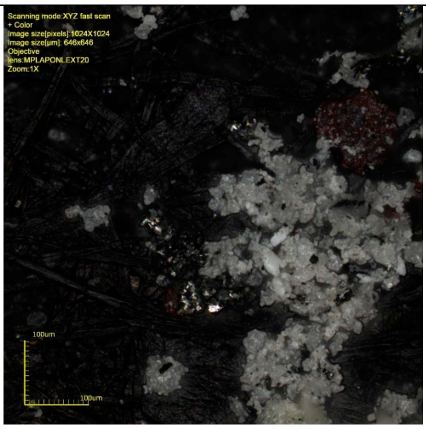	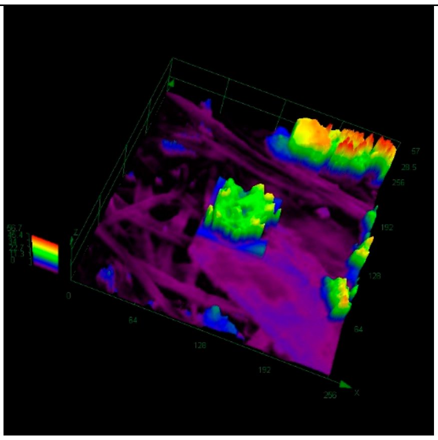

Figure S1. - Confocal laser scanning microscopy images
